# Structure and character analysis of cotton *response regulator* genes family reveals that *GhRR7* responses to draught stress

**DOI:** 10.1186/s40659-022-00394-2

**Published:** 2022-08-16

**Authors:** Lanjie Zhao, Lixue Guo, Xuke Lu, Waqar Afzal Malik, Yuexin Zhang, Jing Wang, Xiugui Chen, Shuai Wang, Junjuan Wang, Delong Wang, Wuwei Ye

**Affiliations:** Institute of Cotton Research of Chinese Academy of Agricultural Sciences/Zhengzhou Research Base, State Key Laboratory of Cotton Biology, School of Agricultural Sciences, Zhengzhou University, Anyang, 455000 Henan China

**Keywords:** Cotton, Response regulators, Abiotic stresses, TCSs

## Abstract

**Background:**

Cytokinin signal transduction is mediated by a two-component system (TCS). Two-component systems are utilized in plant responses to hormones as well as to biotic and abiotic environmental stimuli. In plants, *response regulatory* genes (*RRs*) are one of the main members of the two-component system (TCS).

**Method:**

From the aspects of gene structure, evolution mode, expression type, regulatory network and gene function, the evolution process and role of *RR* genes in the evolution of the cotton genome were analyzed.

**Result:**

A total of 284 *RR* genes in four cotton species were identified. Including 1049 orthologous/paralogous gene pairs were identified, most of which were whole genome duplication (WGD). The *RR* genes promoter elements contain phytohormone responses and abiotic or biotic stress-related cis-elements. Expression analysis showed that *RR* genes family may be negatively regulate and involved in salt stress and drought stress in plants. Protein regulatory network analysis showed that RR family proteins are involved in regulating the DNA-binding transcription factor activity (COG5641) pathway and HP kinase pathways. VIGS analysis showed that the *GhRR7* gene may be in the same regulatory pathway as *GhAHP5* and *GhPHYB*, ultimately negatively regulating cotton drought stress by regulating POD, SOD, CAT, H_2_O_2_ and other reactive oxygen removal systems.

**Conclusion:**

This study is the first to gain insight into *RR* gene members in cotton. Our research lays the foundation for discovering the genes related to drought and salt tolerance and creating new cotton germplasm materials for drought and salt tolerance.

**Supplementary Information:**

The online version contains supplementary material available at 10.1186/s40659-022-00394-2.

## Introduction

Cytokinins are N6-substituted adenine derivatives that have significant functions in various aspects of plant growth and development [1]. Cytokinin signal transduction is mediated by a two-component system (TCS) [2]. Two-component systems are utilized in plant responses to hormones as well as to biotic and abiotic environmental stimuli [3–5]. Related TCSs that have been reported, such as the sensory protein EnvZ that regulates osmotic pressure and the transcription factor OmpR in *E. coli* [6], DegSU that responds to changes in salt concentration found in *Bacillus subtilis* [7] and the temperature-regulated CorSR found in *Pseudomonas syringae* (the biosynthesis of crown toxins is controlled by temperature) [8]. In plants, a two-component system (TCS) is composed of sensor histidine kinases (HKs), histidine phosphotransfer proteins (HPs) and response regulators (RRs) [9]. With the completion of *Arabidopsis* genome sequencing, 32 response regulators (ARRs) have been found in *Arabidopsis* [3]. They all contain a conserved signal-receiving region that can accept phosphate groups. According to their homology, structure and whether their self-expression is induced by cytokinin, the response regulators are roughly divided into four types: A-ARRs, B-ARRs, C-ARRs and *Arabidopsis* pseudoresponse regulators (APRRs) [10–12]. In in vitro experiments, A-ARRs can obtain phosphate groups from AHPs and thus are confirmed to be involved in the binary component signal transduction process. A-ARRs act as negative regulators in the cytokinin signalling pathway [13, 14]. B-ARRs are divided into three types, I, II and III, which act upstream of A-ARRs. B-ARRs act as transcription factors to activate the cytokinin primordial response gene transcription of A-ARRs and other downstream target genes [13–15]. B-ARRs are positive regulators of the cytokinin response [13]. The structure of C-ARRs is similar to that of A-ARRs and contains a signal-receiving region. C-ARRs may be involved in the signal transduction pathway of cytokinins as negative regulators. APRRs lack Asp phosphorylation sites. Studies have shown that some APRRs play a role in regulating circadian rhythms [16]. A study found that *ARR1* plays a critical role in cold signalling and that *AHP2*, *AHP3* and *AHP5* are redundantly involved in cold signalling as positive factors [17]. The expression of *ARR4* and *ARR5* also was sensitive to environmental stresses such as drought, salt and low temperature [18], suggesting a molecular link between stress and cytokinin signalling. Nitrate application also activated *ARR3* through *ARR9* expression [19, 20], presumably due to the elevation of cytokinin levels by nitrate [21]. Response regulators also play a certain role in biological clock-mediated ABA signal transduction. The *Arabidopsis* circadian clock core protein PRRs interact with the key transcription factor ABI5 in the ABA signalling pathway and promote its transcriptional function, thereby coordinating the regulation of ABA signal transduction during seed germination [22].

In other plants, *RR* genes family have also have been reported to respond. *OsRR22* has recently been implicated in cytokinin signalling and metabolism. In *rice*, by the MutMap method [23] to rapidly identify a loss-of-function mutation responsible for the salt tolerance of *hst1* in rice, a B-type response regulator designated *OsRR22* with a Tos17 insertion in the homozygous state indicated that the loss-of-function mutation in *OsRR22* is responsible for the *hst1* salinity tolerance phenotype [24]. The three *maize* genes *ZmPRR73*, *ZmPRR37* and *ZmPRR59* are homologous to *AtPRR7*, *AtPRR3* and *AtPRR9* in *Arabidopsis*, respectively, and have been confirmed to be candidate genes in the *maize* core oscillator [25]. Under cold treatment conditions, a transgenic *maize* population that overexpressed more than 700 *maize* genes in the inbred line LH244 was screened, and a *ZmRR1* an encoding A-type response regulator was identified. Overexpression of *ZmRR1* can significantly improve the cold tolerance of *maize* seedlings, and the *zmrr1-c1/2* mutant obtained by CRISPR/Cas9 is more sensitive to low temperature stress, indicating that *ZmRR1* positively regulates the cold tolerance of maize [26]. In *chickpeas*, *CaRR13* has been found to interact with the promoter of the early nodulation gene *CaNSP2*. Experiments have shown that it acts as a transcription factor that regulates early nodulation. Overexpression of the *CaRR13* gene and complementary RNAi and *cre1* mutant experiments revealed its key role as an important signalling molecule regulating the organogenesis of chickpea nodules [27]. To date, *RR* genes have been identified at a genome-wide scale in various plant species, including *Arabidopsis* [3, 28], rice [12, 29–32], *cabbage* [9], *Lotus japonicas* [33], *soybean* [34, 35], *maize* [25, 36] and *Physcomitrella patens* [37–39]. The *RR* gene in cotton has not been identified, and knowledge of its potential functions in stress adaptations remains confined to *Arabidopsis*, *Chinese cabbage* and rice. The completion of the genome sequencing of cotton allows us to comprehensively identify and analyze the *RR* gene family in cotton [40–44].

In this study, the *RR* gene family derived from the genomic data of cotton species was identified by bioinformatics methods. Gene structural characteristics, chromosomal location, phylogenetic relationships and expression profiles are explained to highlight potential functional diversity. Through RNA-seq data, qRT-PCR and virus silencing expression analysis of its role in salt tolerance and drought resistance, this research will enhance our understanding of the *RR* gene family and the role of *RR* genes in abiotic stresses of cotton, especially drought stress. This laid the molecular foundation for creating new disease-resistant materials and the cultivation of unique varieties of cotton.

## Results

### Identification of *RR* genes in cotton

The RR amino acid sequences reported in *Arabidopsis* and rice were used as query sequences. A total of 93, 94, 48, 49, and 27 genes were confirmed as *RR* family members in *G. hirsutum* (*Gh*), *G. barbadense* (*Gb*), *G. raimondii* (*Gr*), *G. arboreum* (*Ga*) and *Theobroma cacao* (*Tc*). The cotton and *Theobroma cacao RR* genes were named according to their location on chromosomes (Additional file 1: Table S2). The number of *RR* genes in the two allotetraploid cotton lines was almost twice that in the two diploid cotton lines. Their number also was more than that of other plant species (Table. 1), indicating that the *RR* gene family in cotton has undergone expansion during evolution. The number of amino acids, molecular weight (MW) and isoelectric point (pI) were calculated based on the predicted protein sequences. All 284 genes in cotton encoded proteins ranging from 826 (GhRR91) to 70 (GrRR3) amino acids, with protein pIs varying from 10.36 (GrRR3) to 4.58 (GhRR67) and MWs varying from 88.16 (GhRR91) kDa to 8.42 (GrRR3) kDa (Additional file 1: Table S1). The subcellular localization prediction of cotton response regulator proteins showed that most of the Type-A RR and Type-B RR proteins were located in the nucleus, and a few were located in the cytoplasm and extracellular space. The Type-C RRs of the four cotton cultivars were all located in the cytoplasm, chloroplast and nucleus. There are a total of 23 Type-B PRR proteins in cotton, all of which are located in the nucleus. In addition to localization in the nucleus, Type-Clock PRR also is found in organelles such as chloroplasts and mitochondria. These results indicate that cotton response regulators not only participate in biological processes in the nucleus but also participate in cell activities in the cytoplasm and organelles.

**Table 1:** Summary of the *RR* gene numbers identified in plants

Species	Type-A RR	Type-B RR	Type-C RR	Pseudo-RR	Total	References
*Arabidopsis thaliana*	10	12	2	9	33	[3]
*Oryza sativa*	13	13	2	8	36	[12]
*Lotus japonicus*	7	11	1	5	24	[33]
*Glycine max*	18	15	3	13	49	[34]
*Zea mays*	16	9	3	11	39	[25, 36]
*Physcomitrella patens*	7	5	2	4	18	[37–39]
*Brassica rapa*	21	17	4	15	57	[9]
*Theobroma cacao*	6	10	4	7	27	
*G. hirsutum*	22	36	8	27	93	
*G. barbadense*	20	36	9	29	94	
*G. arboreum*	11	20	5	13	49	
*G. raimondii*	10	18	4	16	48	

### Phylogenetic analysis of *RR* genes family in cotton

**Fig. 1:**
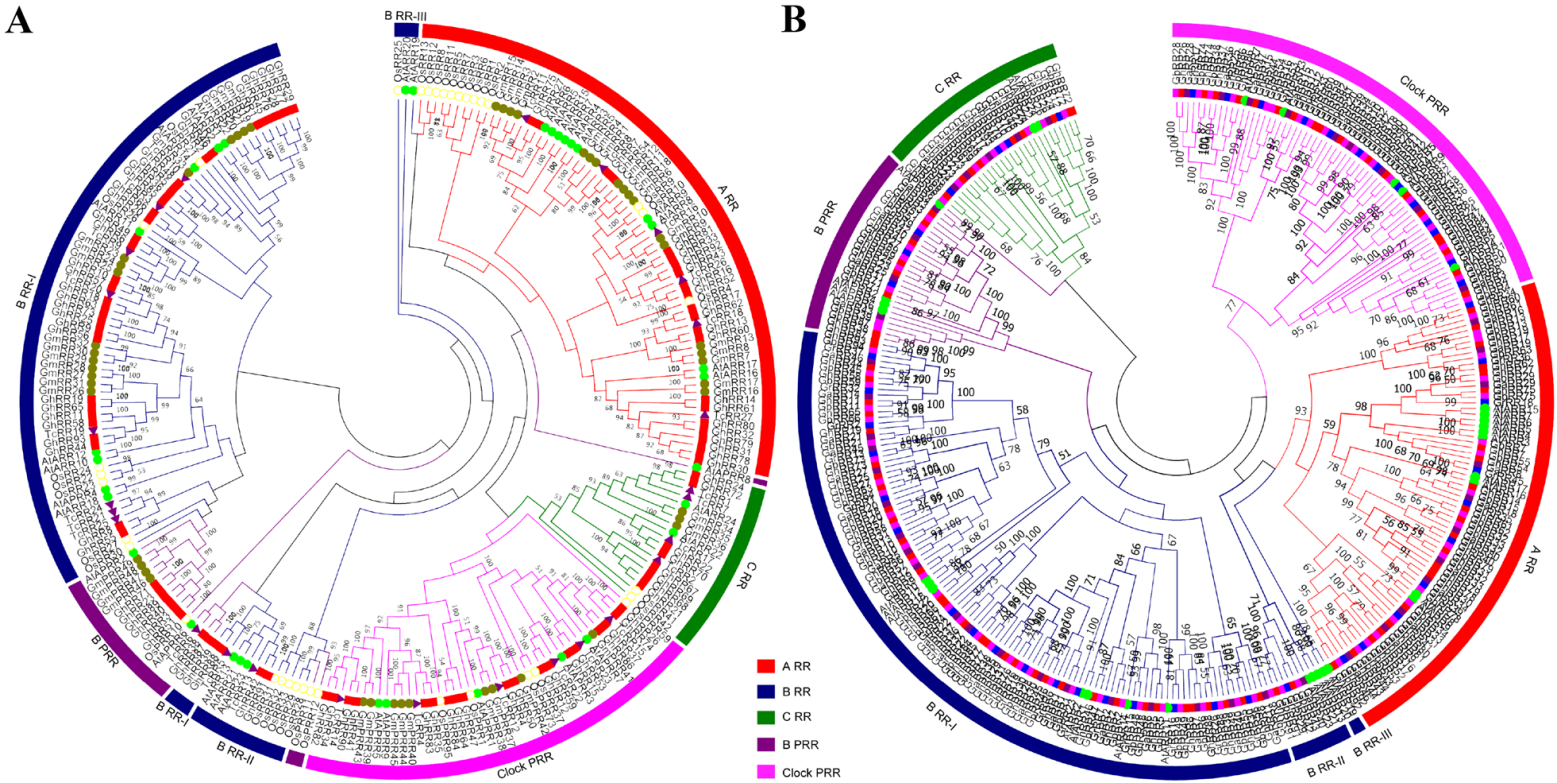
The phylogeny trees of *RR* genes family. **A** Phylogenetic relationship of the 238 identified RR genes from 5 plant species. *Theobroma cacao*, *Arabidopsis thaliana*, *G. hirsutum*, *Glycine max* and *Oryza sativa*. **B** Phylogenetic relationship of the 317 identified *RR* genes from four cotton species and *Arabidopsis*. The two neighbor joining (NJ) phylogeny trees constructed using MEGA 7.0 software. Bootstrap values above 50% from1000 replicates are shown at each node

To investigate the evolutionary relationships among the *RR* genes from four *Gossypium* species, *Theobroma cacao*, *Arabidopsis thaliana*, *Glycine max* and *Oryza sativa*, we constructed a phylogenetic tree in MEGA 7.0 using the NJ method. We utilized 238 orthologous and paralogous RR protein sequences from *Theobroma cacao*, *Arabidopsis thaliana*, *G. hirsutum*, *Glycine max* and *Oryza sativa* to determine the evolutionary history (Fig. 1A). The phylogenetic tree showed that the plant *RR* gene family could be grouped into five subfamilies, namely, *Type-A RR*, *Type-B RR*, *Type-C RR*, *Type-B PRR* and *Type-Clock PRR*, where *Type-B RR* is divided into three subfamilies, i.e., *Type-B RRI*, *Type-B RRII* and *Type-B RRIII* (Fig. 1A).

The largest clade was *Type-B RR* (Table 1). These results demonstrated that the *Type-B RR* subfamily is an ancient group of *RR* genes having the highest number of RR members from almost all plant species. All the subfamilies are composed of monocot and dicot species. Based on the phylogenetic tree, we found that *RR* genes in *G. hirsutum* were more closely related to those in T*heobroma cacao* than other plant species because they always naturally clustered closely to each other in the phylogenetic tree. In most cases, one *Theobroma cacao RR* gene corresponded to two homologous *RR* genes in *G. hirsutum*. Statistics found that some *Theobroma cacao RR* genes corresponded to at least two *G. hirsutum RR* genes, some corresponded to four, some corresponded to six, and at most one *cocoa RR* gene corresponded to eight cotton *RR* genes. For example, *TcRR27* and *GhRR14*, *GhRR61*, *GhRR80*, *GhRR32*, *GhRR79*, *GhRR31*, *GhRR78* and *GhRR30* in *Type-A RR* are in one branch, *TcRR20* and *GhRR10*, *GhRR57*, *GhRR18*, *GhRR63*, *GhRR73*, *GhRR25*, *GhRR9* and *GhRR56* in *Type-B RR* are in one branch, and *TcRR6* and *GhRR71*, *GhRR23*, *GhRR89*, *GhRR40*, *GhRR3*, *GhRR51*, *GhRR38* and *GhRR87* in *Type-B PRR* are in one branch. This shows that ancient cotton experienced a double multiplication event when separated from *grapes* and *cocoa*, forming an ancient tetraploid. After cotton polyploidization, the genome is unstable, resulting in a large number of chromosome rearrangements, DNA segmental inversions and DNA segmental losses, such as *Type-B RRI* branches *GhRR29*, *GhRR77*, *GhRR28*, *GhRR76*, *GhRR45*, *GhRR1*, *GhRR46* and *GhRR2*, which do not correspond to *Theobroma cacao* but cluster with *Glycine max*. Compared to the other dicot species, the phylogenetic analysis showed that the gene families of *G. hirsutum* and *G. barbadense* have experienced significant expansion because the number of *RR* genes of *G. hirsutum* and *G. barbadense* is almost more than double the number of *RR* genes, and due to the conserved functions, they tend to cluster into the same subgroup. In addition, it is a *Type-B RR* response regulator designated *OsRR22* with a Tos17 insertion in the homozygous state, indicating that the loss-of-function mutation in *OsRR22* is responsible for the *hst1* salinity tolerance phenotype [24]. *GhRR10*, *GhRR57*, *GhRR63*, *GhRR93,* GhRR44, etc., in the *Type-B RR* family have the closest homology relationship with *OsRR22*, and they are in the same evolutionary branch. This indicates that they may have the same function and might be used as candidate genes for responsible salinity tolerance.

To investigate the evolutionary and orthologous relationships among different cotton species, the NJ phylogenetic tree was constructed using protein sequences of the cotton RR and AtRR families (Fig. 1B). On the basis of the previous *AtRR* gene family, the phylogenetic tree of all cotton RR proteins also was grouped into five clearly defined subfamilies, each of which contained proteins from *Arabidopsis*, both the diploid and allotetraploid cotton species. In almost every orthologous group, there is one copy in diploid cotton species and two copies in allotetraploid cotton species (one is from the *Arabidopsis* subgenome and the other is from the Dt subgenome). The clustering results further confirmed that two allotetraploid cotton species, *G. hirsutum* and *G. barbadense*, were the result of hybridization and doubled between the ancestors of two diploid cotton species, *G. arboreum* and *G. raimondii* [42].

### Chromosomal localization of cotton *RR* genes

To clearly understand the chromosome distribution of the *RR* gene family, we drew a chromosome distribution map of 284 cotton *RR* gene family members (Additional file 2: Fig. S1). The results show that in *G. arboreum*, 49 *RR* genes are distributed on the remaining 12 chromosomes except for chr02, of which chr05 has the most genes, with a total of eight *GaRR11*, *GaRR12*, *GaRR13*, *GaRR14*, *GaRR15*, *GaRR16*, *GaRR17* and *GaRR18* genes*.* The *RR* genes were evenly distributed on 13 chromosomes in *G. raimondii*. In *G. hirsutum* and *G. barbadense*, except for AD1-A03 and AD2-A02, no *RR* gene distribution and all other chromosomes were distributed, and the *RR* genes distributed on the fifth chromosome were the most abundant. This shows that the *RR* genes were duplicated after the fifth chromosome was doubled in the genome. In addition, there were paired parallel homologous genes between the corresponding subgenomes of the *GhRR* and *GbRR* family genes in the A subgenome and the D subgenome. In AD1-A10 and AD1-D10, and in AD1-A11 and AD1-D11, there were five pairs of parallel homologous genes in the four pairs of subgenomes, AD2-A12, AD2-D12, AD1-A12 and AD1-D12. AD1-A01 and AD1-D01, and AD1-A13 and AD1-D13, are two pairs of subgenomes. There were two pairs of parallel homologous genes in AD1-A06 and AD1-D06, and in AD2-A06 and AD2-D06, there was one pair of parallel homologous genes in the two pairs of subgenomes, AD2-A08 and AD2-D08, and AD2-A010, and there were four pairs of parallel homologous genes in the two pairs of subgenomes of AD2-D10. There were three pairs of parallel homologous genes in the pair of subgenomes AD2-A09 and AD2-D09.

### Collinearity analysis for cotton *RR* genes

**Fig. 2:**
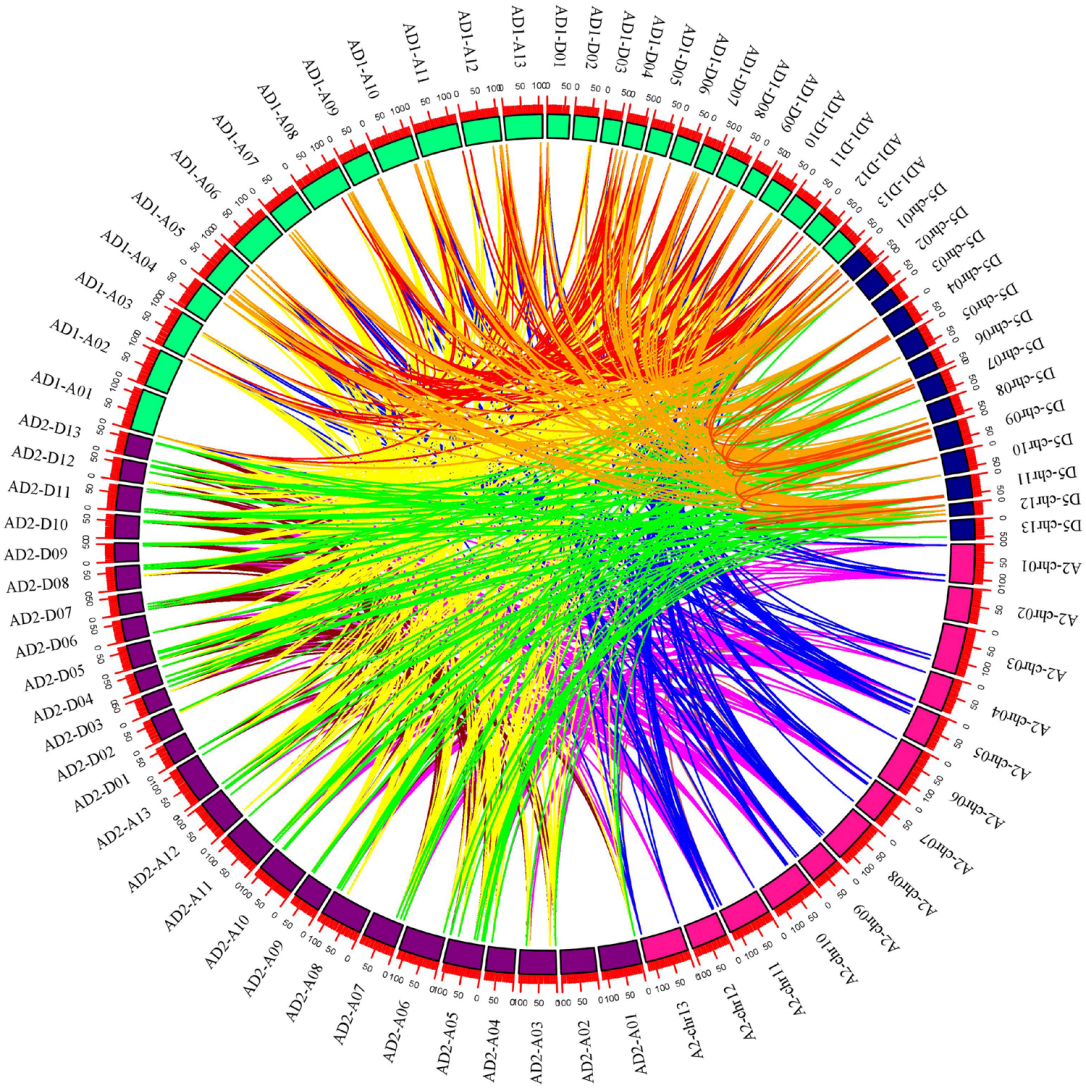
Syntenic relationships among *RR* genes of two diploid (*G. arboreum* and *G. raimondii*) and two allotetraploid (*G. hirsutum* and *G. barbadense*) cotton. The chromosomes of *G. arboreum*, *G. raimondii*, *G. hirsutum* and *G. barbadense* were shown with pink, blue, green and Purple colors, respectively

The gene family refers to the genes derived from the same ancestor, a set of genes composed of two or more copies produced. The evolution of gene families arises through three processes, namely, whole-genome duplication, segmental duplication and tandem duplication [56]. The evolutionary relationships between the *RR* gene families of diploid and tetraploid cotton species were evaluated based on chromosomal distances, coverage and resemblance between their members, which was used to identify segmental tandem repeats and whole-genome duplication. The genes that lie on the same chromosomal block (e-value < 1e−5) were categorized as tandemly duplicated, while the remaining genes from the same genomes were considered segmental; other genes from different genomes and subgenomes of four *Gossypium* species were allocated in whole genome duplication [57]. Combined analysis of *RR* genes from *G. hirsutum*, *G. barbadense*, *G. arboreum* and *G. raimondii* was performed to analyze the gene duplications and syntenic relationships among them. *G. barbadense* (*Gb*) and *G. hirsutum* (*Gh*) genes were duplicated between *G. raimondii* (*Gr*) and *G. arboreum* (*Ga*), which indicates the origination of both tetraploid genomes from diploid genomes (A and D) during polyploidization (Additional file 1: Table S2). A total of 1049 orthologous/paralogous gene pairs were identified, of which 239 pairs were predicted in segmental duplication to form paralogous gene pairs within the GhAt/GhDt, GbAt/GbDt, A2/A2 and D5/D5 subgenomes, while 810 orthologous gene pairs experienced whole genome duplication. No tandem duplication gene pairs were found for *RR* genes family. From these results, we presumed that orthologous/paralogous gene pairs were generally raised from WGD before polyploidization was involved in the evolutionary process (Fig. 2).

### Structure analysis of cotton *GhRRs*

**Fig. 3:**
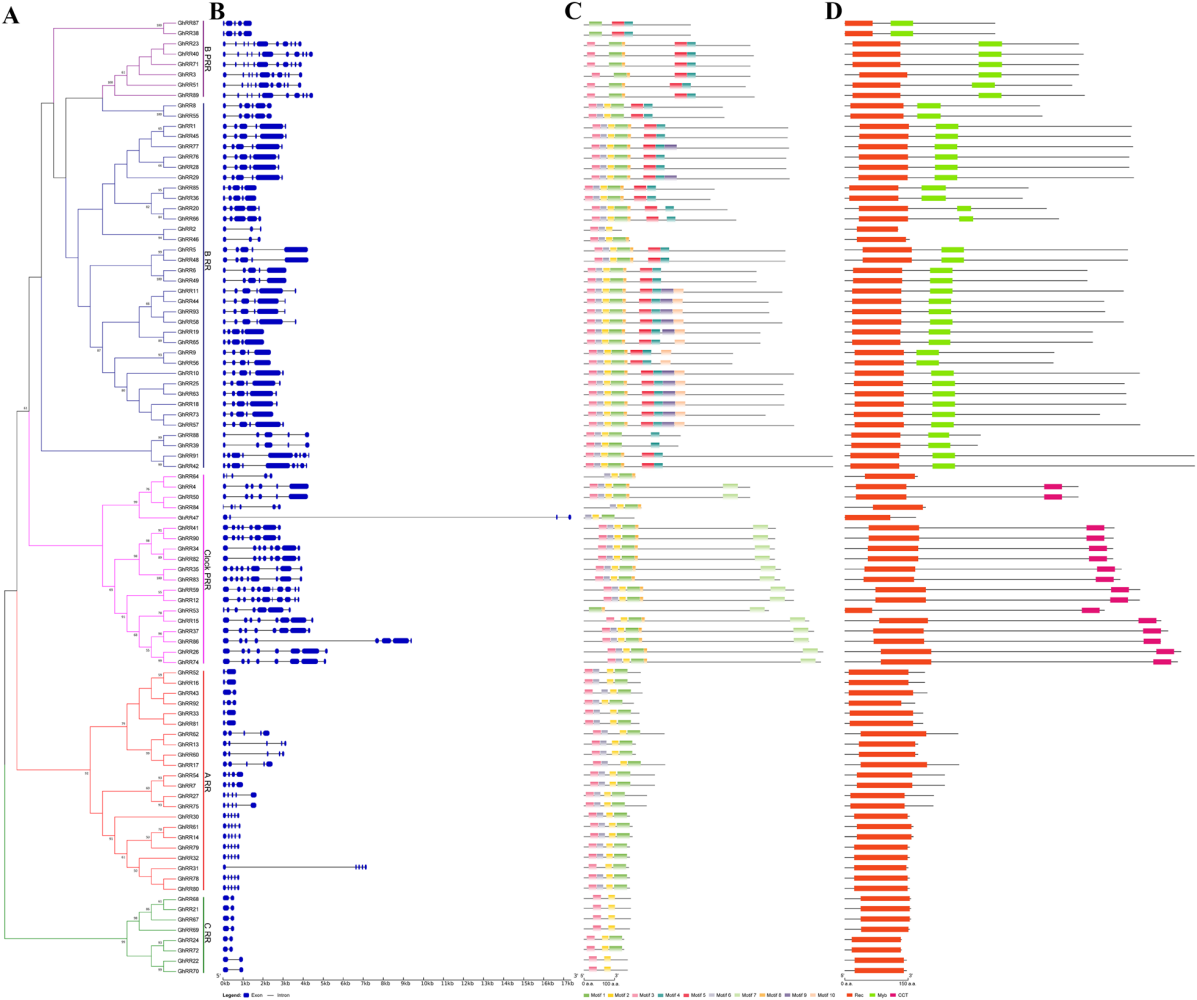
Comparison of the gene structure, conserved protein motifs and domains in *RR* genes on the *G. hirsutum*. **A** The NJ phylogenetic tree was constructed based on the full-length sequences of *G. hirsutum* RR proteins using MEGA 7.0 software. Details of subfamilies are shown in different colors. **B** Exon–intron structure of *G. hirsutum RR* genes. Blue boxes indicate exons; black lines indicate introns. **C** The motif composition of *G. hirsutum*. The motifs, numbers 1–10, are displayed in different colored boxes. **D** Schematic representation of the conserved domains in *G. hirsutum* RR proteins. The Rec domains, Myb and CCT domains are highlighted by red boxes, green boxes and watermelon red boxes, respectively. The length of DNA genomic or protein can be estimated using the scale at the bottom

We analyzed the gene structure, motif and protein domains of the *RR* genes identified in upland cotton (Fig. 3). Exon/intron analysis and phylogenetic tree results show that the *RR* gene families all have exons and introns, and genes in the same subfamily usually have surprisingly similar exon/intron structures. Except for *GhRR47* in the *Type-Clock PRR* subfamily and *GhRR56* and *GhRR31* in the *Type-A RR* subfamily, the three gene intron sequences are longer than those in the same subfamily (Fig. 3B). These results indicate that genes with different gene structures may have special biological functions. A total of 10 motifs were identified by the online program MEME and named Motif 1–10. As shown in Fig. 3C, except for Motif 1, which is a widely distributed RR domain, as expected, the same subfamily of *RR* gene members usually has a common motif, indicating that their functions are similar. We also found that some protein motifs existed in multiple members of the five classes. However, others are specific to a particular class. For example, 10 motifs exist only in one branch of the *Type-B RR* type. In addition to the three motifs of *GhRR47* and *GhRR56*, *Type-C RR* has only two motifs for the rest of the genes. The *Type-A RR* gene contains only four motifs. The unique motifs in different subfamilies may represent the conservation and specific functions of the *GhRR* gene family (Fig. 3C).

**Fig. 4:**
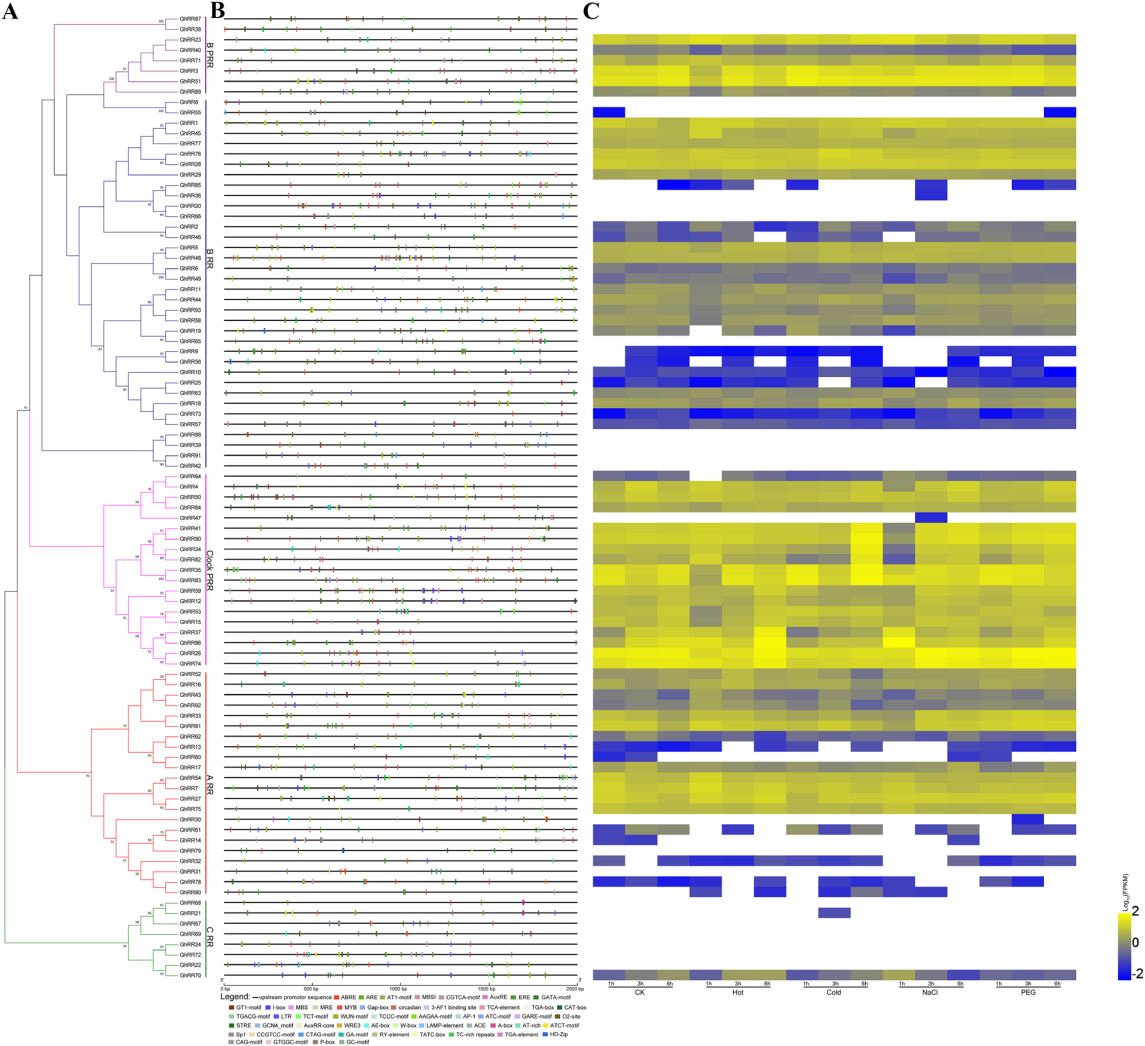
Analysis of *RR* genes promoter and its expression pattern under different stresses. **A** Phylogenetic tree of GhRRs. **B** Cis-elements in promoters of *GhRRs*. **C** Expression pattern of *GhRRs* under different stresses

Through the analysis of protein domains, it was found that 93 *GhRR* family gene members all have response regulator receiver domains. In addition, Type-Clock PRR not only contains a response regulator receiver domain but also contains a unique CCT motif on the C side. The CCT (CONSTANS, CO-like and TOC1) domain is a highly conserved basic module of 43 amino acids that is found near the C-terminus of plant proteins and is often involved in light signal transduction. The CCT domain contains a putative nuclear localization signal within the second half of the CCT motif and has been shown to be involved in nuclear localization and probably also has a role in protein–protein interaction [58]. Type-B PRRs and Type-B RRs also have a special MYB-like DNA-binding domain.

### Promoter cis-elements analysis of *GhRRs* and their roles in gene expression under different abiotic stresses

The cis-acting element regulates gene expression and is closely related to the biological function of genes. We selected a 2000 bp 5′-flanking region upstream of the transcription start site of each *RR* gene in *G. hirsutum* and identified and analyzed them with the PlantCARE database. At the same time, we performed analysis of differentially expressed genes under cold, heat, salt and PEG stress for various durations (1 h, 3 h and 6 h) using RNA-seq. In the 93 *RR* gene promoter regions, 30 types of growth and development cis-elements, 14 types of phytohormone response cis-elements and nine types of abiotic or biotic stress cis-elements were identified (Fig. 4; Additional file 1: Table S4). It is worth noting that among the nine cis-elements of abiotic or biotic stress, analysis of 15 *RR* gene promoter cis-elements revealed that the MYB binding site was involved in drought inducibility. For example, *GhRR78* and *GhRR92* are classified as *Type-A RRs*, *GhRR23*, *GhRR51* and *GhRR71* are classified as *Type-B PRRs*, and *GhRR50* and *GhRR82* are classified as *Type-Clock PRRs*. These seven genes are all highly expressed under RNA-Seq stress. There were also 116 MYB motifs (which play a vital role in the regulation of auxin-regulated genes), 82 ABRE motifs (involved in abscisic acid responsiveness), 41 CGTCA motifs (regulatory elements involved in MeJA responsiveness), 32 LTRs (involved in low-temperature responsiveness), 37 W-boxes (wounding and pathogen response sites), 22 TC-rich repeats (involved in defence and stress responsiveness) and 122 EREs (ethylene-responsive elements) in the *RRs* that were highly expressed under other stresses (Fig. 4C).

### Tissue expression patterns of *RR* genes in upland cottons

Through the analysis of the transcriptome data of different tissues in upland cotton (root, stem, leaves, flowers, ovules and fiber) [51] (Additional file 3: Fig. S2; Additional file 1: Table S7), the expression patterns in each subfamily were basically the same, and the expression patterns between subfamilies were quite different. However, most of the *RR* genes present a constitutive expression pattern. A small number of genes, such as the *Type-C RR* subfamily, were expressed at low levels or even not in the tissue group. The expression levels of the *Type-Clock PRR* subfamily genes in the initial stages of root and fibre development (0 DPA, 1 DPA) were significantly higher than those of other subfamilies. *Type-Clock PRR* subfamily members *GhRR4*, *GhRR50*, *GhRR37*, *GhRR86*, *GhRR26* and *GhRR74* have high expression levels in various tissues and cotton fibre development stages. This result implies that the abovementioned genes have unique functions and are relatively conserved during the evolution of cotton. This phenomenon also can be found in the *Type-B RR* branch.

### Expression analysis of *RR* genes under salt stress and drought stress

**Fig. 5:**
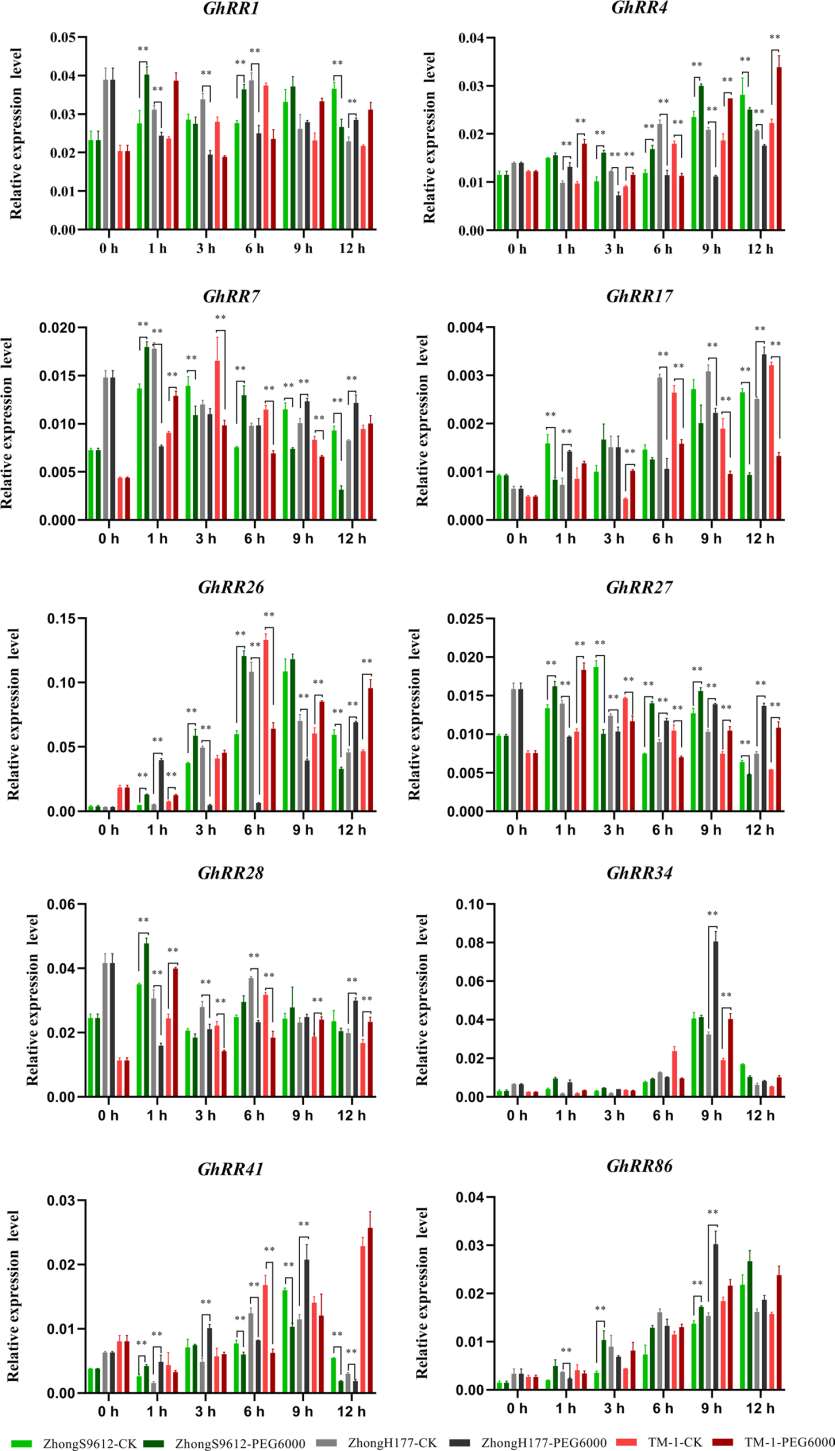
Expression levels of 10 *GhRRs* in 12% PEG6000 drought stresses. Error bars represent SD of three independent experiments

**Fig. 6:**
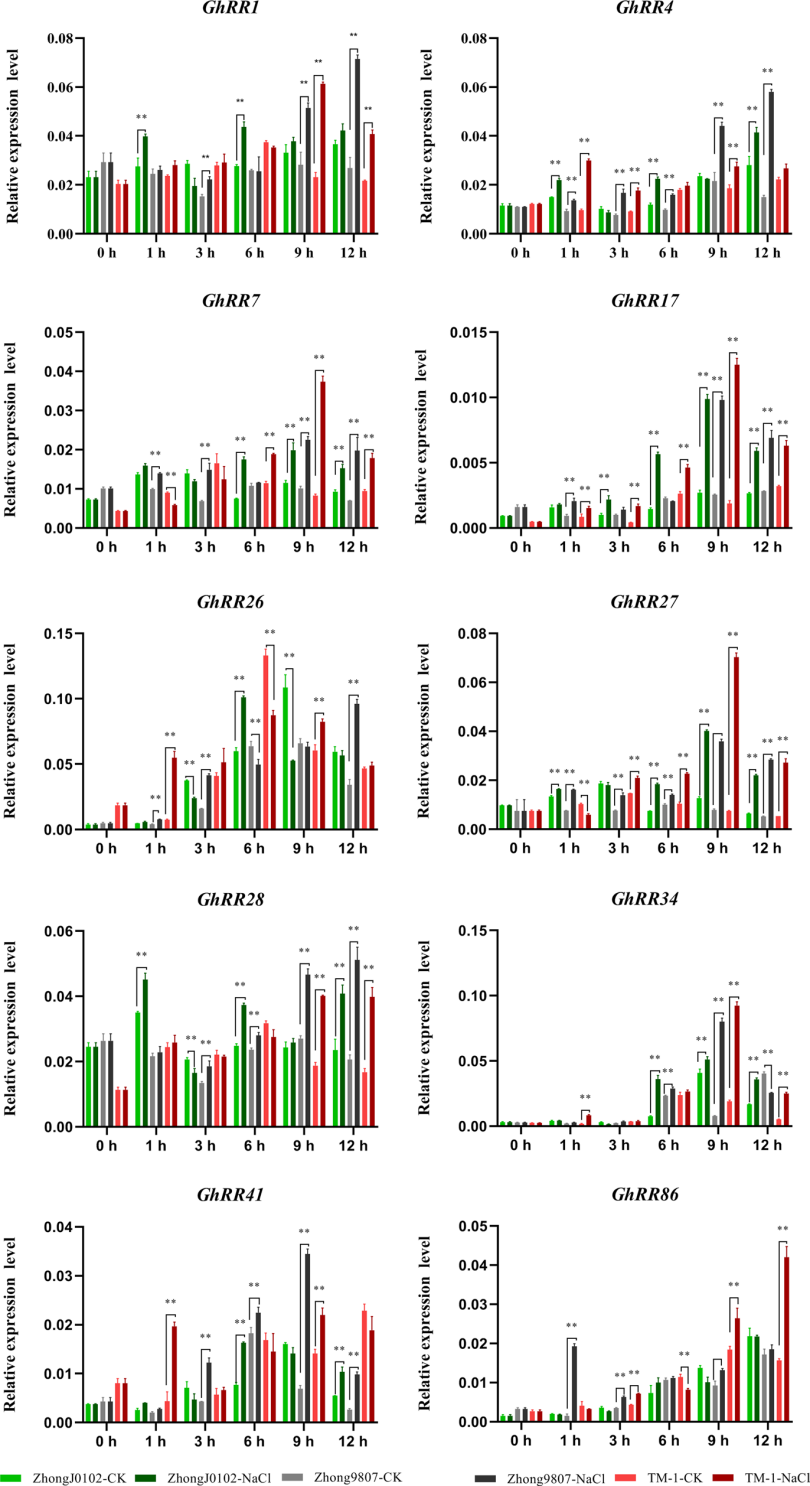
Expression levels of 10 *GhRRs* in 400 mM NaCl stresses. Error bars represent SD of three independent experiments

To further ascertain whether *GhRR* gene family expression levels were related to abiotic stress, referring to the functional analysis of the rice *RR *gene family, *Arabidopsis* and other crops have been studied for *RR* gene function, with RNA-seq data for abiotic stress analysis. We selected three *Type-A RR* genes, *GhRR7*, *GhRR17* and *GhRR27*, three *Type-B RR* genes, *GhRR1*, *GhRR4* and *GhRR28*, and four *Type-Clock PRR* genes, *GhRR26*, *GhRR34*, *GhRR41* and *GhRR86*, for a total of 10 genes as our research objects. Real-time fluorescence quantitative expression analysis was performed. To analyze the expression types of the selected genes more clearly and intuitively, we selected five varieties: salt-tolerant material Zhong9807, salt-sensitive material ZhongJ0102, drought-tolerant material ZhongH177, drought-sensitive material ZhongS9612 and genetics standard TM-1. We detected the relative expression levels of 10 genes at different time periods after treatment with 12% PEG6000 (Fig. 5) and 200 mM NaCl (Fig. 6).

The qPCR results showed that the expression levels of each gene in different time periods and different materials were different. The expression trend of each gene under salt stress and drought stress also was inconsistent. The expression level of *GhRR1* in salt-sensitive materials after 1 h and 6 h treatments was significantly higher than that of the control. In salt-tolerant materials, with the prolonged stress time, the expression level showed a trend of first decline and then increase, and the expression level was significantly lower at 3 h. Compared with the control, the expression at 9 h and 12 h showed a very significant difference. In the standard line TM-1, the expression level of *GhRR1 first* increased and then decreased, reached the highest level at 9 h, and decreased slightly at 12 h, but it was significantly higher than that of the control. The expression level of *GhRR1* after drought treatment was similar to that after salt treatment, and the expression level was the lowest at 3 h, which was consistent with the abiotic stress RNA-Seq data of *GhRR1*. In contrast to *GhRR1*, after salt treatment, the expression level of *GhRR4* showed an upwards trend in salt-tolerant materials. After drought treatment, the expression level showed a trend of first declining and then increasing. Regardless of salt stress or drought stress, *GhRR4* showed an upwards trend in salt-sensitive materials. The material is the same as the expression in TM-1, showing a fluctuating state. Under salt treatment, the expression of *GhRR7* in the salt-tolerant materials increased significantly at 3 h, and the expression decreased in the salt-sensitive materials. The expression trends of the three materials were basically the same in the other three time periods. In contrast, after drought treatment with *GhRR7*, except for the expression of the three materials at 3 h, the expression levels of salt-tolerant materials and salt-sensitive materials were always opposite, and the expression level of TM-1 fluctuated. This indicates that *GhRR7* may be involved in the regulation of drought tolerance in cotton.

Under salt stress, the expression patterns of *GhRR17* and *GhRR27* were the same. As the amount of time increases, the expression trends of the three materials are consistent. At 1 h and 12 h after drought treatment, the expression levels of drought-sensitive and drought-tolerant materials were significantly opposite. Quantitative data show that *GhRR17* also may participate in cotton drought regulation. In contrast to the previous genes, *GhRR26* had different expression trends in different materials under salt stress and drought stress. The trends of salt-tolerant materials and salt-sensitive materials were the same at 9 h, but the other time periods were significantly opposite. In drought-tolerant and drought-sensitive materials, the expression trend remained constant at 1 h, and the expression trends in other time periods also showed a significant opposite trend. It is speculated that *GhRR26* not only participates in the regulation of cotton drought tolerance but also participates in the regulation of cotton salt tolerance. Under salt stress, the expression trend of *GhRR28* was the same for the salt-tolerant and salt-sensitive materials at 3 h. Under drought stress, the expression level of *GhRR28* under drought-tolerant materials showed at first a decline and then an upwards trend, while under drought-sensitive materials, the expression level of *GhRR28* showed an expression trend that first increased and then decreased. Compared with photos, there was a very significant difference, which indicates that *GhRR28* may respond to drought stress in the early stage of stress. The expression level of *GhRR34* was not high under drought stress and salt stress in the early stage, and the expression trend in each material was basically the same. The expression trend of *GhRR41* under drought stress and salt stress was basically the same, except that the expression trend of salt-tolerant materials and salt-sensitive materials was opposite except at 9 h. It is speculated that *GhRR41* may participate in cotton salt stress and drought stress after stress. Under salt stress, the expression of *GhRR41* showed the same trend in salt-tolerant and salt-sensitive materials. Under drought stress, the expression trends of *GhRR86* were drought-sensitive materials and drought-tolerant materials at 1 h and 3 h, and the expression trends were the same in the later period, which indicated that *GhRR86* might be involved in cotton drought regulation in the early stage of drought stress. This is basically the same as the heat map made by RNA-Seq data.

### Protein interaction network

**Fig. 7:**
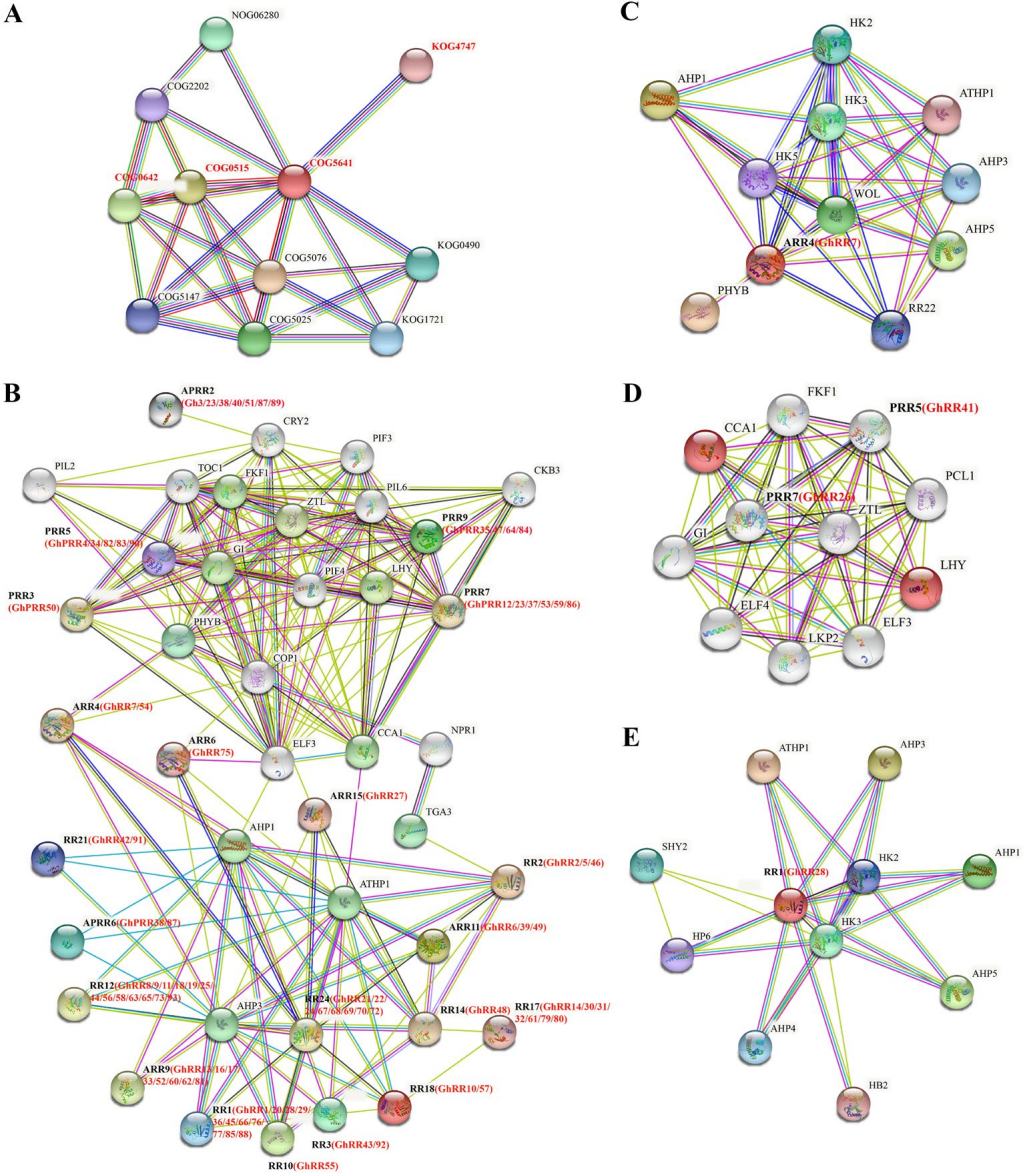
Interaction network of RR proteins. **A** Interaction network of RR proteins families. The red letters represent RR proteins signaling pathway. **B** Interaction network of GhRRs proteins with other proteins. The black and red letters represent AtRR proteins and cotton RR proteins, respectively. **C** Interaction network of GhRR7 proteins with other proteins. The black and red letters represent AtRR proteins and GhRR7 proteins, respectively. **D** Interaction network of GhRR41 and GhRR26 proteins with other proteins. The black letters represent AtRR proteins, red letters represent GhRR41 and GhRR26 proteins, respectively. **E** Interaction network of GhRR28 proteins with other proteins. The black and red letters represent AtRR proteins and GhRR28 proteins, respectively

To analyze the function of the cotton RR proteins, we used gene homology to analyze the protein interaction network of the *RR* genes in *Arabidopsis thaliana* through software. Through the fully studied *Arabidopsis* RR proteins, we can infer the large part of the regulatory network involved in cotton RR proteins [54, 59] (Fig. 7). performing a protein family search, we found the DNA-binding transcription factor activity (COG5641) of cotton RR proteins and other related regulatory pathways, such as participation in plant drought, high salt, low temperature, hormones (ET/ABA/GA/auxin), trauma, serine/threonine protein kinase signal transduction mechanisms [60] for pathogen response and cell cycle regulation, and other signal transduction processes. Two-component phosphor lay intermediate involved in MAP kinas cascade regulation and signal transduction histamine kinase. This shows that the function of cotton RR members depends on the two-component signal transduction system and serine/threonine protein kinase signal transduction.

By multiple sequence searches (Fig. 7B), all Type-C RR proteins interacted with AHP family proteins. Deserving of special attention is that almost all Type-B PRRs interact with other proteins by interacting with the CRY2 (cryptochrome-2) protein. In *Arabidopsis*, CRY2 can perceive low blue light (LBL) and respond by directly contacting two bHLH transcription factors, PIF4 and PIF5, at chromatin on E-box variant 5'-CA[CT]GTG-3' to promote their activity and stimulate specific gene expression to adapt global physiology (e.g., hypocotyl elongation and hyponastic growth in low blue light) [61]. In response to blue light irradiation, it triggers the nuclear accumulation of ROS and finally regulates plant abiotic stress. It is speculated that the cotton *Type-B PRR* gene also has the function of responding to abiotic stress. In addition, through the multisequence search method, we also found that GIGANTEA protein (GI), phytochrome interaction factor 7 (PIF7), phytochrome A (PHYA) and phytochrome B (PHYB) proteins participate in circadian clock signal regulation through light conversion. *Arabidopsis* histidine phosphotransfer protein (AHP) is involved in the phosphate relay signal transduction of histidine (His) to aspartate (Asp) [62].

We found that the proteins that interact with GhRR7 are mainly histidine kinases such as WOL (histidine kinase 4) [63], *Arabidopsis thaliana* histidine phosphotransfer proteins (AHPs) [64] and phytochrome B (PHYB) [62], which participate in the photoconversion and induce an array of morphogenetic responses. This implies that the GhRR7 protein not only participates in the regulation of the histidine protein kinase pathway but also participates in the transfer of light signals. Unlike GhRR7, GhRR28 not only interacts with AHPs but also with nonsymbiotic haemoglobin 2 (HB2), which may not function as an oxygen storage or transport protein but might act as an oxygen sensor or play a role in electron transfer, possibly to a bound oxygen molecule. GhRR28 has a low affinity for O(2), belongs to the plant globin family, and interacts with the UX/IAA transcriptional regulator family protein SHY2 in a regulatory pathway. Aux/IAA proteins are short-lived transcription factors that function as repressors of early auxin response genes at low auxin concentrations [65]. This indicates that the GhRR28 protein is involved in more regulation in plants. Both GhRR41 and GhRR26 belong to the Type-Clock PRR subfamily. Through protein comparison, we found that *Arabidopsis* PRR5 and PRR7, which are in the same branch as the GhRR41 and GhRR26 proteins in the phylogenetic tree, passed the homeodomain-like superfamily protein PCL1 [66] and LHY [67], Galactose oxidase/kelch repeat superfamily protein ZTL [68], protein EARLY FLOWERING 4 (ELF4) [69], LOV KELCH PROTEIN2 (LKP2) [68], CIRCADIAN CLOCK ASSOCIATED 1 (CCA1) [70], FLAVIN-BINDING, KELCH REPEAT, F-BOX 1 (FKF1) [71] and other proteins involved in the interaction of plant biological clock regulation and function. It is speculated that cotton GhRR41 and GhRR26 proteins also are involved in regulating the response function of the cotton circadian clock.

### Drought tolerance of cotton enhanced after *GhRR7* gene silencing

**Fig. 8:**
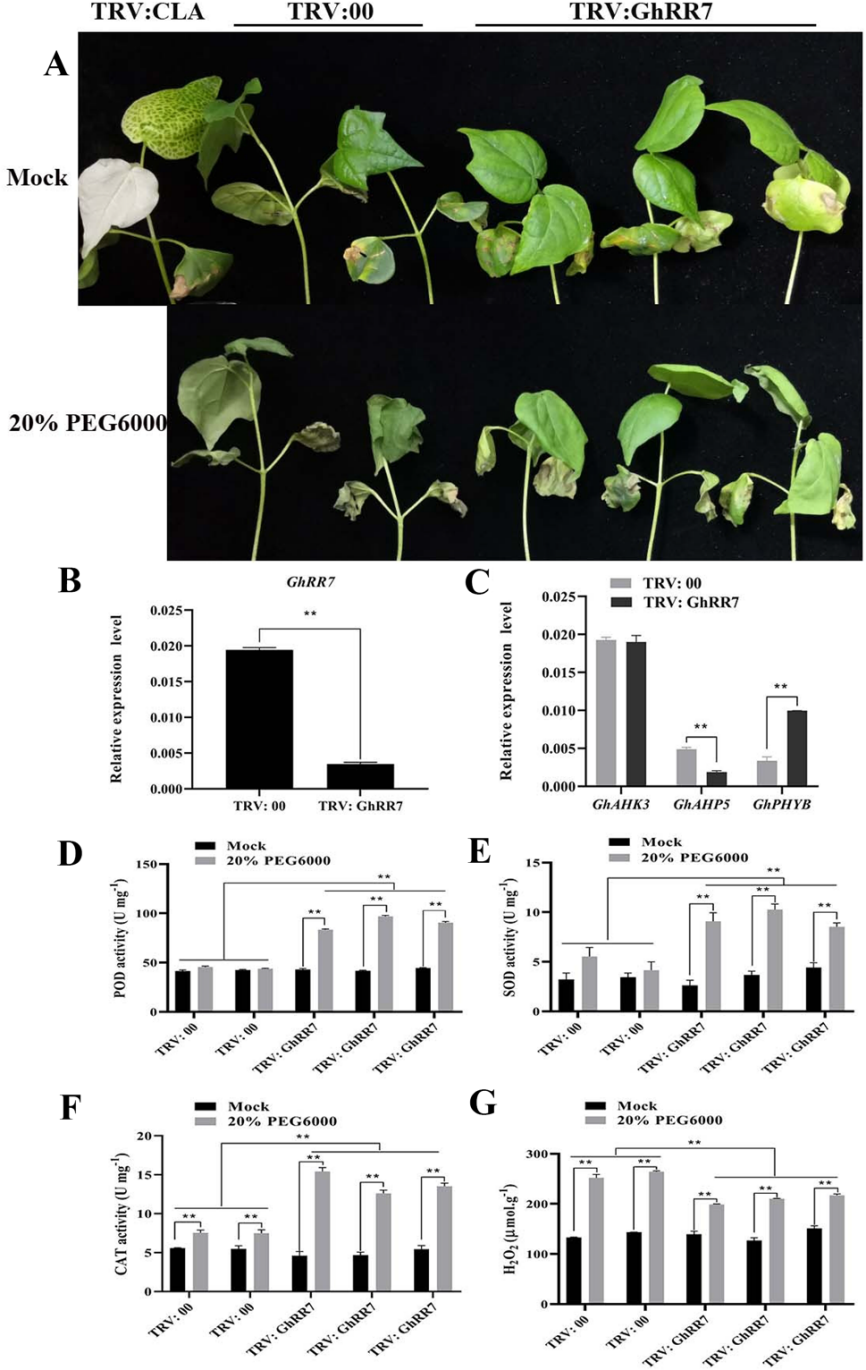
Silencing of *GhRR7* compromised cotton drought stress tolerance. **A** The positive control plants, control and target gene-silenced before 20% PEG6000 stress treatment. **B** Relative expression levels of control plants and target gene-silenced. **C** The expression levels of *GhAHK3*, *GhAHP5* and *GhPHYB* genes in *GhRR7* silenced plants. **D** POD activity between control plants and target gene-silenced under mock and 20% PEG6000 stress treatment. **E** SOD activity between control plants and target gene-silenced under mock and 20% PEG6000 stress treatment The expression levels of *GhAHK3*, *GhAHP5* and *GhPHYB* genes in *GhRR7* silenced plants. **F** CAT activity between control plants and target gene-silenced under mock and 20% PEG6000 stress treatment. **G** H_2_O_2_ amount of control plants and target gene-silenced under mock and 20% PEG6000 stress treatment. The data represent the means_SE from three independent experiments, t-tests: *p < 0.05, **p < 0.01

Combined with the fluorescence quantitative results and RR protein interaction network, we selected the *GhRR7* gene for further research. Based on the interaction network analysis and differential expression patterns under different abiotic stresses, we hypothesized that *GhRR7* is potentially important in the regulation of stress responses. To verify our hypothesis, we adopted a VIGS approach to knock down the expression of *GhRR7* using TRV vectors, TRV: GhRR7. TRV: CLA was used as the positive control (Fig. 8A).

Two weeks after VIGS, when albino seedlings appeared in the positive control, qRT-PCR was used to determine the expression levels in the leaves of the TRV: *GhRR7* and TRV: 00 control plants. The results show that the transcript levels of *GhRR7* were significantly reduced following two weeks of VIGS (Fig. 8B). We treated the empty and silent plants with 20% PEG6000 and found that the silent plants exhibited a phenotype of wilting and water loss. In the next 12 h, the control plants (TRV: 00) displayed severe wilting and yellowing of leaves compared to the target gene-silenced plants under drought stress. Plants have a complete antioxidant enzyme system, which can eliminate ROS generated after drought stress. These antioxidant enzyme activities can directly reflect the stress resistance of plants. We tested the activity of POD, SOD, CAT and contents of H_2_O_2_ in the target gene-silenced plants and control plants (Fig. 8D–G). The results showed that compared with control plants, the activity of POD, SOD and CAT in the target gene-silenced plants’ enzyme activity were significantly increased, the contents of H_2_O_2_ in the arget gene-silenced plants were significantly depressed compared with those in the TRV: 00 plants, thereby increasing the removal of ROS and improving the drought tolerance of cotton.

To further explore the function of the GhRR7 protein, we selected three TCS system genes, *GhAHK3*, *GhAHP5* and *GhPHYB*, to design specific primers (Additional file 1: Table S1) based on the predicted results of the GhRR7 protein regulatory network. The expression levels of three genes in the TRV: *GhRR7* target gene-silenced plants were detected (Fig. 8C). The results showed that, compared with control plants, the expression levels of the *GhAHK3* genes did not change significantly. The expression level of *GhAHP5* in the target gene-silenced plants was significantly lower than that in the control plants, and the expression level of *GhPHYB* in the target gene-silenced plants was significant. It shows that *GhRR7* may regulate the expression of downstream *GhAHP5* gene and participate in the response of cotton to drought stress.

## Discussion

*RR* genes have been identified at a genome-wide scale in various plant species, and knowledge of their potential functions in stress adaptations remains confined to *Arabidopsis* [3, 17]*, Chinese cabbage* [9] and rice [12, 23]. *RR* genes in cotton have not been identified, and few studies have been performed on their functions in cotton. By using AtRR proteins as queries, we identified 93, 94, 48 and 49 *RR* gene family members in *G. hirsutum*, *G. barbadense*, *G. raimondii* and *G. arboreum*, respectively. The phylogenetic tree analysis showed that cotton *RR* genes belonged to four categories: *Type-A RR*, *Type-B RR*, *Type-C RR* and *pseudo-RR*. Two allotetraploid cotton species, *G. hirsutum* and *G. barbadense*, were the result of hybridization and doubled between the ancestors of two diploid cotton species, *G. arboreum* and *G. raimondii*. Type-B RR response regulators designated as *OsRR22* mutation *hst1* salinity tolerance phenotype [24] and *GhRR10*, *GhRR57*, *GhRR63*, *GhRR93* and *GhRR44*, etc., in *Type-B RR* genes family have the closest homology relationship with *OsRR22*. This indicates that they may have the same function and might be used as candidate genes for responsible salinity tolerance.

Through the analysis of chromosome location and collinearity, we found that the *RR* genes in cotton were unevenly distributed in the chromosomes, and there was no distribution in A2-chr02, AD1-A03 and AD2-A02. In cotton, a total of 1049 orthologous/paralogous gene pairs were identified; 239 pairs were predicted in segmental duplication to form paralogous gene pairs within the GhAt/GhDt, GbAt/GbDt, A2/A2 and D5/D5 subgenomes, while 810 orthologous gene pairs experienced whole genome duplication. No tandem duplication gene pairs were found for *RR* genes family.

Through the analysis of *GhRR* gene structure and motif and protein domains, we found that different types of *GhRR* genes have different numbers of introns and exons, and motifs also are specific, but *GhRR* genes all contain response regulator receiver domains. Type-clock PRRs not only contain a response regulator receiver domain but also contain a unique CCT motif on the C side. The CCT domain contains a putative nuclear localization signal within the second half of the CCT motif and has been shown to be involved in nuclear localization and probably also has a role in protein–protein interaction [58]. Type-B PRRs and Type-B RRs also have a special MYB-like DNA-binding domain.

Through elemental analysis of *GhRR* gene promoters and RNA-Seq data analysis, a total of 30 types of growth and development cis-elements, 14 types of phytohormone response cis-elements, and nine types of abiotic or biotic stress cis-elements were identified. The *GhRR* genes not only play a role in plant growth and development but also may participate in the regulation of plant hormones and abiotic stress. Analysis of 15 *RR* gene promoter cis-elements revealed that the MYB binding site was involved in drought inducibility. There were also 116 MYB motifs (which play a vital role in the regulation of auxin-regulated genes), 82 ABRE motifs (involved in abscisic acid responsiveness), 41 CGTCA motifs (regulatory elements involved in MeJA responsiveness), 32 LTRs (involved in low-temperature responsiveness), 37 W-boxes (wounding and pathogen response sites), 22 TC-rich repeats (involved in defence and stress responsiveness) and 122 EREs (ethylene-responsive elements) in the *RR* genes that were highly expressed under other stresses (Fig. 5C). Through the analysis of the transcriptome data of different tissues of upland cotton (root, stem, leaves, flowers, ovules and fibres) [51] (Fig. 6; Additional file 1: Table S5), most of the *RR* genes presented a constitutive expression pattern. A small number of genes were low or even not expressed in the tissue group. This result implies that the abovementioned genes have unique functions and are relatively conserved during the evolution of cotton. This phenomenon also can be found in the *Type-B RR* branch.

To further ascertain whether *GhRR* gene family expression levels were related to abiotic stress, with RNA-seq data for abiotic stress analysis, we selected three *Type-A RR* genes, *GhRR7*, *GhRR17* and *GhRR27*, three *Type-B RR* genes, *GhRR1*, *GhRR4* and *GhRR28*, and four *Type-Clock PRR* genes, *GhRR26*, *GhRR34*, *GhRR41* and *GhRR86*, for a total of 10 genes as the research objects. The experimental results show that under drought and salt stress, the expression levels of *GhRR* genes have different levels compared with the control up- or downregulation.

Protein interaction network analysis showed that DNA-binding transcription factor activity (COG5641) was found around the interaction network, which participates in plant drought, high salt, low temperature, hormones (ET/ABA/GA/auxin), trauma, serine/threonine protein kinase signal transduction mechanisms [60] for pathogen response and cell cycle regulation, and other signal transduction processes. This shows that the function of cotton *RR* gene members depends on the two-component signal transduction system and serine/threonine protein kinase signal transduction. By analyzing the interacting proteins of the three proteins GhRR7, GhRR28 and GhRR41, we found that the GhRR7 and GhRR28 proteins are involved in the regulation of AHP signal transduction in the two-component signal transduction system, while GhRR41 is mainly related to the plant biological clock and light signal transduction. This result is consistent with previous studies [22, 64].

To further explore the role of the *GhRR7* gene in the regulation of cotton drought tolerance, we constructed a TRV: *GhRR7* silencing vector. The results showed that with 20% PEG6000 treatment, the leaves of the control plants wilted obviously, while the phenotype of the silent plants did not change significantly. These antioxidant enzyme activities can directly reflect the stress resistance of plants. We tested the activity of POD, SOD, CAT and contents of H_2_O_2_ in target gene-silenced plants and control plants. The results showed that compared with the control plants, the enzyme activity of POD,SOD and CAT in the target gene-silenced plants was significantly increased, the contents of H_2_O_2_ in the arget gene-silenced plants were significantly depressed compared with those in the TRV: 00 plants, thereby increasing the removal of ROS and improving the salt tolerance of cotton. Based on the results of GhRR7 protein regulatory network analysis, we speculated that GhRR7 may be in the same regulatory pathway as GhAHP5 and GhPHYB. The expression levels of the three genes in TRV: *GhRR7* target gene silenced plants were examined (Fig. 8E), which indicated that the expression of *GhRR7* is consistent with that of *GhAHP5* but opposite that of *GhPHYB*, and *AHP5* histidine phosphotransfer proteins function as redundant negative regulators of the drought stress response, as also has been reported in *Arabidopsis* [72]. *PHYB* can improve drought tolerance by enhancing ABA sensitivity in *Arabidopsis* [73], which is consistent with our results.

## Conclusions

In summary, 93, 94, 48 and 49 RR family members in *G. hirsutum*, *G. barbadense*, *G. raimondii* and *G. arboreum*, respectively, were identified. This study is the first to gain insight into *RR* gene members in cotton. From the aspects of gene structure, evolution mode, expression type, regulatory network and gene function, the evolution process and role of *RR* genes in the evolution of the cotton genome were analyzed. Cotton is an important crash crop, and drought is one of the important reasons that limits its yield and quality. Our research lays the foundation for discovering the genes related to drought and salt tolerance and creating new cotton germplasm materials for drought and salt tolerance.

## Materials and methods

### Databases

The four cotton genome files *Gossypium arboreum* (CRI, version 1.0), *G. raimondii* (JGI, version 2.0), *G. hirsutum* (ZJU, version 1.0) and *G. barbadense* (ZJU, version 1.0) were downloaded from the Cotton Functional Genomics Database (CottonFGD) (https://cottonfgd.org/) [44]. The genome sequences of *Oryza sativa* (version 10), *Theobroma cacao* (version 10), *Glycine max* (version 10) and *Arabidopsis thaliana* were retrieved from JGI (https://phytozome.jgi.doe.gov/pz/portal.html).

### Identification of cotton *RR* genes family members

We downloaded the hidden Markov model (HMM) (version 3.0) profile of PF00072 from Pfam (https://pfam.xfam.org/). Then, we used HMMER 3.0 software (http://www.hmmer.org/) with default parameters and settings to acquire the *RR* genes of Pfam PF00072, which most likely belongs to the *RR* gene family. We further evaluated our genes by using (https://pfam.xfam.org/) Pfam and (http://smart.emblheidelberg.de/) SMART (Simple Modular Architecture Research Tool) for confirmation of results. Finally, we further confirmed the identified RRs manually. We also retrieved some other features of RRs, such as upland cotton-like isoelectric points (pIs), molecular weights (MWs), exon/intron lengths, grand average of hydropathy, and charge by using the Cotton Functional Genomic Database (CottonFGD) (http://www.cottonfgd.org/) [44].

### Phylogenetic analysis and sequences alignments

The full-length amino acid sequences of *G. hirsutum*, *G. arboreum*, *G. raimondii*, *G. barbadense*, *Arabidopsis thaliana*, *Theobroma cacao*, *Glycine max* and *Oryza sativa* encoded by *RR* genes were aligned with the ClustalW program (version 2.0) with the default settings and then manually adjusted in MEGA 7.0. Subsequently, we constructed the neighbour joining (NJ) tree with 1000 bootstrap replicates, using the Poisson substitution model with default parameters in MEGA 7.0 [45].

### Chromosomal locations of *RR* genes from four *Gossypium* species

The physical positions of chromosomal locations from four cotton species, including *G. hirsutum*, *G. arboreum*, *G. raimondii* and *G. barbadense,* were drawn with the help of TBtools software 9 [46]. The genomic sequences, CDSs and sequences of all four species were downloaded from the Cotton Functional Genomic Database (CottonFGD) (http://www.cottonfgd.org/) [44] and their genome assembly sequences from CottonGen (https://www.cottongen.org) [47].

### Collinearity analysis of *RR* genes in four *Gossypium* species

Syntenic relationships between duplicated gene pairs from four cotton species, *G. hirsutum*, *G. arboreum*, *G. raimondii* and *G. barbadense*, were analyzed by using MCScanX software [48], and diagrammatical results were visualized by using simple Circos-0.69 software (http://circos.ca/) [49]. Tandem duplicated *RR* genes were identified when they belonged to the same subfamily but had a separation gap of 10 or fewer genes within 200 kb.

### Analysis of conserved protein motifs and gene structure

We used the Multiple EM for Motif Elicitation (MEME) website (http://meme-suite.org/) to identify the conserved protein motifs [50]. The figure of the phylogenetic tree along with the gene structure and conserved protein motifs was drawn with TBtools software [46] using the MAST file from the MEME website, the NWK file from phylogenetic tree analysis and the GFF3 genome file of *G. hirsutum*. The CDS and genomic sequences of the *G. hirsutum* genome were used to draw a picture of exon/intron organization at the Gene Structure Display Server (GSDS) program (http://gsds.cbi.pku.edu.cn/).

### Analysis of *GhRR* promoter regions and differentially expressed genes

DNA sequences of 2000 bp upstream of of the transcription start site of *GhRRs* were obtained from the CottonFGD database (http://www.cottonfgd.org/) as promoters [44]. We used the PlantCARE website (http://bioinformatics.psb.ugent.be/webtools/plantcare/html/) for the prediction of cis-regulatory elements in promoter region *GhRR* genes. Cis-acting elements related to phytohormones, plant growth and development, and abiotic stress were selected for further analysis. We used RNA-Seq data (PRJNA248163) downloaded from the National Center for Biotechnology Information (NCBI) (https://www.ncbi.nlm.nih.gov/) to analyze differentially expressed genes under salt, PEG, cold and heat stresses [51]. The heat map, along with the phylogenetic tree and cis-acting elements, was generated through TBtools software using fragments per kilobase of exon per million mapped (FPKM).

### Subcellular localization of GhRRs in upland cotton

Subcellular localization prediction of GhRRs was carried out by using several websites, such as TargetP (http://www.cbs.dtu.dk/services/TargetP/) [52], CELLO v.2.5 (http://cello.life.nctu.edu.tw/) [53], WoLF PSORT (https://wolfpsort.hgc.jp/) and ProtComp (http://linux1.softberry.com/berry.phtml?topic=protcomppl&Group=proGrams&subGroup=proloc).

### Interaction network of GhRR proteins

STRING software (https://string-db.org/) [54] was used to analyze the interaction among RR proteins on the basis of the orthologues in *Arabidopsis* with a confidence parameter set at the 0.4 threshold.

### Plant material and treatment for expression analysis

Upland cotton material “Zhong9807, ZhongJ0102, TM-1, ZhongH177, ZhongS9612” was obtained from Institute of Cotton Research of Chinese Academy of Agricultural Sciences. Seeds of Zhong9807 (salt insensitive), ZhongJ0102 (salt sensitive), TM-1 (genetic standards), ZhongH177 (drought insensitive) and ZhongS9612 (drought sensitive) accessions were grown in chambers at a controlled 25 °C temperature for 16 h/8 h day/night. Zhong8907 roots, stems and leaves were taken at the three-leaf stage, rapidly placed in liquid nitrogen, and stored at − 80 °C after preservation. To determine the expression patterns of *GhRRs* under different stress conditions, leaves of plants exposed to 12% PEG6000 and 200 mM NaCl at the three-leaf stage were collected for RNA extraction at 1 h, 3 h, 6 h, 9 h and 12 h separately. Plants treated with water were considered as controls. Total RNA was isolated by using an EASYspin Plus Plant RNA quick isolation Kit (Aidlab Co., LTD, Beijing, China). The pure RNA was reverse-transcribed using the PrimeScript™ RT reagent Kit with gDNA Eraser (Takara Biomedical Technology Co., LTD, Beijing, China) according to the manufacturer's instructions. Specific primers for qPCR were designed using NCBI (https://www.ncbi.nlm.nih.gov/tools/primer-blast/index.cgi?LINK_LOC=BlastHomewebsite). All primer sequences are shown Additional file 1: Table S1. qRT-PCR was performed using the Bio-Rad CFX96 fluorescence quantitative PCR platform with TB Green^®^ Fast qPCR Mix (Takara Biomedical Technology Co., LTD, Beijing, China) in accordance with the manufacturer's protocol. Each sample was collected as three independent biological replicates. The relative gene expression levels were calculated based on the 2^−ΔΔCT^ method [55]. The cotton *histone 3* gene (GenBank accession No.AF024716) was used as a standard control.

### Vector construction and procedure for VIGS in cotton

Virus-induced gene silencing (VIGS) in cotton followed by pathogen inoculation-TRV vectors and *Agrobacterium tumefaciens* for VIGS were prepared. Inserts to generate TRV: *GhRR7* and positive control TRV: *CLA* were amplified from the cDNA of *Gossypium hirsutum *L. Zhong9807. Primer pairs to generate TRV vectors are shown in Additional file 1: Table S1. PCR fragments were digested with *Bam*HI and *Sac*I and then ligated into TRV: 00. The products were transformed into *A. tumefaciens* GV3101. TRV vectors were agroinfiltrated as described into the cotyledons of 7-day-old seedlings of Zhong9807. The seedlings were then grown at 25 °C with a 16 h/8 h light/dark photoperiod cycle in a controlled environment chamber. After verifying the VIGS efficiency through qRT-PCR, the roots of both control and target gene-silenced plants were irrigated with 20% PEG6000 as drought stress up to 12 h [74]. The VIGS experiments were repeated with three replicates, and 15 plants were used during each replication.

### Determination of drought stress-related physiological parameters

The superoxide dismutase (SOD) activity and peroxidase (POD) activity of TRV: 00 and TRV: *GhRR7* were determined by the POD activity detection kit (Solarbio, BC0170, Beijing, China) and the SOD activity detection kit (Solarbio, BC0090). The catalase (CAT) activities was quantified from 100 mg cotton leaves using a CAT assay kit as previously described [75]. The amount of H_2_O_2_ was measured spectrophotometrically using a standard curve prepared with the known concentrations of H_2_O_2_ [76].

## Supplementary Information

**Additional file 1: Table S1.** The primers used for experiment in this study. **Table S2.** Information of *RR* genes in different species. **Table S3.** Information of the cotton *RR* genes in this study. **Table S4.** Collinearity analyses of *RR* genes family in four cotton species. **Table S5.** Analysis of *GhRRs* cis-elements. **Table S6.** RNA-Seq data analysis of *GhRRs* expression profiling in different stresses. **Table S7.** RNA-Seq data analysis of *GhRRs* expression profiling in different tissues.

**Additional file 2: Figure S1.** Chromosomal location of four *Gossypium* species. The scale on the left is inmega-bases. The gene ID on the right side of each chromosome corresponds to each *RR* gene's approximate locations. (A) *Gossypium arboreum* A-genome “A2”. (B) *Gossypium raimondii* D-sub genome “D5”. (C) *Gossypium hirsutum* genome “AD1”. (D) *Gossypium barbadense* genome “AD2”.

**Additional file 3: Figure S2.** Analysis of *RR* genes expression pattern in different tissues. (A) Phylogenetic tree of GhRRs. (B) Expression pattern of *GhRRs* in different tissues.

## Data Availability

All the data is contained in the manuscript.

## Abbreviations

RR: Response regulators
TCS: Two-component system
HP: Histidine phosphotransfer proteins
HK: Heterogeneous histidine kinase-like proteins
ABA: Phenylalanine ammonialyase
HMM: Hidden Markov model
pIs: Isoelectric points
MWs: Molecular weights
NJ: Neighbor joining
GSDS: Gene structure display server
VIGS: Virus-induced gene silencing
SOD: Superoxide dismutase
POD: Peroxidase
CAT: Catalase
WGD: Electrospray ionization
*G. arboreum*: *Gossypium arboreum*
ET: Differential expression genes
*G. hirsutum*: *Gossypium hirsutum*
*G. raimondii*: *Gossypium raimondii*
*G. barbadense*: *Gossypium barbadense*
qRT-PCR: Quantitative real-time polymerase chain reaction

## References

[1] Mok DW, Mok MC (2001). Cytokinin metabolism and action. Annu Rev Plant Physiol Plant Mol Biol.

[2] Yamada S, Shiro Y (2008). Structural basis of the signal transduction in the two-component system. Adv Exp Med Biol.

[3] Hwang I, Chen H-C, Sheen J (2002). Two-component signal transduction pathways in *Arabidopsis*. Plant Physiol.

[4] Hwang I, Sheen J, Müller B (2012). Cytokinin signaling networks. Annu Rev Plant Biol.

[5] Hutchison CE, Kieber JJ (2002). Cytokinin signaling in *Arabidopsis*. Plant Cell.

[6] Cai SJ, Inouye M (2002). EnvZ-OmpR interaction and osmoregulation in *Escherichia coli*. J Biol Chem.

[7] Dartois V, Débarbouillé M, Kunst F, Rapoport G (1998). Characterization of a novel member of the DegS-DegU regulon affected by salt stress in *Bacillus subtilis*. J Bacteriol.

[8] Ullrich M, Peñaloza-Vázquez A, Bailey AM, Bender CL (1995). A modified two-component regulatory system is involved in temperature-dependent biosynthesis of the *Pseudomonas syringae* phytotoxin coronatine. J Bacteriol.

[9] Liu Z, Zhang M, Kong L, Lv Y, Zou M, Lu G, Cao J, Yu X (2014). Genome-wide identification, phylogeny, duplication, and expression analyses of two-component system genes in Chinese cabbage (*Brassica rapa *ssp.* pekinensis*). DNA Res.

[10] D'Agostino IB, Deruère J, Kieber JJ (2000). Characterization of the response of the *Arabidopsis response regulator* gene family to *Cytokinin1*. Plant Physiol.

[11] Mason MG, Li J, Mathews DE, Kieber JJ, Schaller GE (2004). Type-B response regulators display overlapping expression patterns in *Arabidopsis*. Plant Physiol.

[12] Schaller GE, Doi K, Hwang I, Kieber JJ, Khurana JP, Kurata N, Mizuno T, Pareek A, Shiu SH, Wu P (2007). Nomenclature for two-component signaling elements of *rice*. Plant Physiol.

[13] To JPC, Kieber JJ (2008). Cytokinin signaling: two-components and more. Trends Plant Sci.

[14] To JPC, Haberer G, Ferreira FJ, Deruère J, Mason MG, Schaller GE, Alonso JM, Ecker JR, Kieber JJ (2004). T*ype-A Arabidopsis response regulators* are partially redundant negative regulators of cytokinin signaling[W]. Plant Cell.

[15] Sakai H, Honma T, Aoyama T, Sato S, Kato T, Tabata S, Oka A (2001). ARR1, a transcription factor for genes immediately responsive to cytokinins. Science.

[16] McClung CR (2006). Plant circadian rhythms. Plant Cell.

[17] Jeon J, Kim J (2013). *Arabidopsis* response regulator1 and Arabidopsis histidine phosphotransfer Protein2 (AHP2), AHP3, and AHP5 function in cold signaling. Plant Physiol.

[18] Urao T, Yakubov B, Yamaguchi-Shinozaki K, Shinozaki K (1998). Stress-responsive expression of genes for two-component response regulator-like proteins in *Arabidopsis thaliana*. FEBS Lett.

[19] Kiba T, Taniguchi M, Imamura A, Ueguchi C, Mizuno T, Sugiyama T (1999). Differential expression of genes for response regulators in response to cytokinins and nitrate in *Arabidopsis thaliana*. Plant Cell Physiol.

[20] Taniguchi M, Kiba T, Sakakibara H, Ueguchi C, Mizuno T, Sugiyama T (1998). Expression of *Arabidopsis* response regulator homologs is induced by cytokinins and nitrate. FEBS Lett.

[21] Takei K, Sakakibara H, Taniguchi M, Sugiyama T (2001). Nitrogen-dependent accumulation of cytokinins in root and the translocation to leaf: implication of cytokinin species that induces gene expression of *maize* response regulator. Plant Cell Physiol.

[22] Yang M, Han X, Yang J, Jiang Y, Hu Y (2021). The *Arabidopsis* circadian clock protein PRR5 interacts with and stimulates ABI5 to modulate abscisic acid signaling during seed germination. Plant Cell.

[23] Abe A, Kosugi S, Yoshida K, Natsume S, Takagi H, Kanzaki H, Matsumura H, Yoshida K, Mitsuoka C, Tamiru MJNB (2012). Genome sequencing reveals agronomically important loci in *rice* using MutMap. Nat Biotechnol.

[24] Takagi H, Oli MT, Abe A, Yoshida K, Uemura A, Yaegashi H, Obara T, Oikawa K, Utsushi H, Kanzaki E (2015). MutMap accelerates breeding of a salt-tolerant *rice* cultivar. Nat Biotechnol.

[25] Dong Z, Danilevskaya O, Abadie T, Messina C, Coles N, Cooper M (2012). A gene regulatory network model for floral transition of the shoot apex in *maize* and its dynamic modeling. PLoS ONE.

[26] Zeng R, Li Z, Shi Y, Fu D, Yin P, Cheng J, Jiang C, Yang S (2021). Natural variation in a type-A response regulator confers *maize* chilling tolerance. Nat Commun.

[27] Tiwari M, Yadav M, Singh B, Pandey V, Nawaz K, Bhatia S (2021). Evolutionary and functional analysis of two-component system in chickpea reveals *CaRR13*, a TypeB RR, as positive regulator of symbiosis. Plant Biotechnol J.

[28] Schaller GE, Kieber JJ, Shiu S-H. Two-component signaling elements and histidyl-aspartyl phosphorelays. Arabidopsis Book. 2008;6. 10.1199/tab.0112 PMC3243373 22303237

[29] Tsai YC, Weir NR, Hill K, Zhang W, Kim HJ, Shiu SH, Schaller GE, Kieber JJ (2012). Characterization of genes involved in cytokinin signaling and metabolism from *rice*. Plant Physiol.

[30] Ito Y, Kurata N (2006). Identification and characterization of cytokinin-signalling gene families in *rice*. Gene.

[31] Pareek A, Singh A, Kumar M, Kushwaha HR, Lynn AM, Singla-Pareek SL (2006). Whole-genome analysis of *Oryza sativa* reveals similar architecture of two-component signaling machinery with *Arabidopsis*. Plant Physiol.

[32] Du L, Jiao F, Chu J, Jin G, Chen M, Wu P (2007). The two-component signal system in rice (*Oryza sativa* L.): a genome-wide study of cytokinin signal perception and transduction. Genomics.

[33] Ishida K, Niwa Y, Yamashino T, Mizuno T (2009). A genome-wide compilation of the two-component systems in *Lotus japonicus*. DNA Res.

[34] Mochida K, Yoshida T, Sakurai T, Yamaguchi-Shinozaki K, Shinozaki K, Tran LS (2010). Genome-wide analysis of two-component systems and prediction of stress-responsive two-component system members in *soybean*. DNA Res.

[35] Le DT, Nishiyama R, Watanabe Y, Mochida K, Yamaguchi-Shinozaki K, Shinozaki K, Tran LS (2011). Genome-wide expression profiling of soybean two-component system genes in *soybean* root and shoot tissues under dehydration stress. DNA Res.

[36] Chu ZX, Ma Q, Lin YX, Tang XL, Zhou YQ, Zhu SW, Fan J, Cheng BJ (2011). Genome-wide identification, classification, and analysis of two-component signal system genes in *maize*. Genet Mol Res.

[37] Pils B, Heyl A (2009). Unraveling the evolution of cytokinin signaling. Plant Physiol.

[38] Ishida K, Yamashino T, Nakanishi H, Mizuno T (2010). Classification of the genes involved in the two-component system of the moss *Physcomitrella patens*. Biosci Biotechnol Biochem.

[39] Satbhai SB, Yamashino T, Okada R, Nomoto Y, Mizuno T, Tezuka Y, Itoh T, Tomita M, Otsuki S, Aoki S (2011). Pseudo-response regulator (PRR) homologues of the moss *Physcomitrella patens*: insights into the evolution of the PRR family in land plants. DNA Res.

[40] Wang K, Wang Z, Li F, Ye W, Wang J, Song G, Yue Z, Cong L, Shang H, Zhu S (2012). The draft genome of a diploid cotton *Gossypium raimondii*. Nat Genet.

[41] Paterson AH, Wendel JF, Gundlach H, Guo H, Jenkins J, Jin D, Llewellyn D, Showmaker KC, Shu S, Udall J (2012). Repeated polyploidization of *Gossypium* genomes and the evolution of spinnable cotton fibres. Nature.

[42] Li F, Fan G, Wang K, Sun F, Yuan Y, Song G, Li Q, Ma Z, Lu C, Zou C (2014). Genome sequence of the cultivated cotton *Gossypium arboreum*. Nat Genet.

[43] Li F, Fan G, Lu C, Xiao G, Zou C, Kohel RJ, Ma Z, Shang H, Ma X, Wu J (2015). Genome sequence of cultivated Upland cotton (*Gossypium hirsutum* TM-1) provides insights into genome evolution. Nat Biotechnol.

[44] Zhu T, Liang C, Meng Z, Sun G, Meng Z, Guo S, Zhang R (2017). CottonFGD: an integrated functional genomics database for cotton. BMC Plant Biol.

[45] Kumar S, Stecher G, Tamura K (2016). MEGA7: molecular evolutionary genetics analysis version 7.0 for bigger datasets. Mol Biol Evol.

[46] Chen C, Xia R, Chen H, He Y (2018). TBtools, a toolkit for biologists integrating various HTS-data handling tools with a user-friendly interface. bioRxiv.

[47] Yu J, Jung S, Cheng C-H, Ficklin SP, Lee T, Zheng P, Jones D, Percy RG, Main D (2014). CottonGen: a genomics, genetics and breeding database for cotton research. Nucleic Acids Res.

[48] Wang Y, Tang H, Debarry JD, Tan X, Li J, Wang X, Lee TH, Jin H, Marler B, Guo H (2012). MCScanX: a toolkit for detection and evolutionary analysis of gene synteny and collinearity. Nucleic Acids Res.

[49] Gascoyne R (2009). Circos: an information aesthetic for comparative genomics. Genome Res.

[50] Bailey TL, Boden M, Buske FA, Frith M, Grant CE, Clementi L, Ren J, Li WW, Noble WS (2009). MEME SUITE: tools for motif discovery and searching. Nucleic Acids Res.

[51] Hu Y, Chen J, Fang L, Zhang Z, Ma W, Niu Y, Ju L, Deng J, Zhao T, Lian J (2019). *Gossypium barbadense* and *Gossypium hirsutum* genomes provide insights into the origin and evolution of allotetraploid cotton. Nat Genet.

[52] Emanuelsson O, Nielsen H, Brunak S, von Heijne G (2000). Predicting subcellular localization of proteins based on their N-terminal amino acid sequence. J Mol Biol.

[53] Yu C-S, Chen Y-C, Lu C-H, Hwang J-K (2006). Prediction of protein subcellular localization. Proteins.

[54] Wang X, Lu X, Malik WA, Chen X, Wang J, Wang D, Wang S, Chen C, Guo L, Ye W (2020). Differentially expressed bZIP transcription factors confer multi-tolerances in *Gossypium hirsutum* L. Int J Biol Macromol.

[55] Schmittgen TD, Livak KJ (2008). Analyzing real-time PCR data by the comparative C(T) method. Nat Protoc.

[56] Xu G, Guo C, Shan H, Kong H (2012). Divergence of duplicate genes in exon-intron structure. Proc Natl Acad Sci USA.

[57] Malik WA, Wang X, Wang X, Shu N, Cui R, Chen X, Wang D, Lu X, Yin Z, Wang J (2020). Genome-wide expression analysis suggests glutaredoxin genes response to various stresses in cotton. Int J Biol Macromol.

[58] Strayer C, Oyama T, Schultz TF, Raman R, Somers DE, Más P, Panda S, Kreps JA, Kay SA (2000). Cloning of the Arabidopsis clock gene *TOC1*, an autoregulatory response regulator homolog. Science.

[59] Zhu S, Wang X, Chen W, Yao J, Li Y, Fang S, Lv Y, Li X, Pan J, Liu C (2021). Cotton *DMP* gene family: characterization, evolution, and expression profiles during development and stress. Int J Biol Macromol.

[60] Xing HT, Guo P, Xia XL, Yin WL (2011). PdERECTA, a leucine-rich repeat receptor-like kinase of poplar, confers enhanced water use efficiency in *Arabidopsis*. Planta.

[61] Pedmale UV, Huang SC, Zander M, Cole BJ, Hetzel J, Ljung K, Reis PAB, Sridevi P, Nito K, Nery JR (2016). Cryptochromes interact directly with PIFs to control plant growth in limiting blue light. Cell.

[62] Jung JH, Domijan M, Klose C, Biswas S, Ezer D, Gao M, Khattak AK, Box MS, Charoensawan V, Cortijo S (2016). Phytochromes function as thermosensors in *Arabidopsis*. Science.

[63] Tran LS, Urao T, Qin F, Maruyama K, Kakimoto T, Shinozaki K, Yamaguchi-Shinozaki K (2007). Functional analysis of *AHK1*/*ATHK1* and cytokinin receptor histidine kinases in response to abscisic acid, drought, and salt stress in *Arabidopsis*. Proc Natl Acad Sci USA.

[64] Tanaka Y, Suzuki T, Yamashino T, Mizuno T (2004). Comparative studies of the *AHP* histidine-containing phosphotransmitters implicated in His-to-Asp phosphorelay in *Arabidopsis thaliana*. Biosci Biotechnol Biochem.

[65] Koren D, Resnick N, Gati EM, Belausov E, Weininger S, Kapulnik Y, Koltai H (2013). Strigolactone signaling in the endodermis is sufficient to restore root responses and involves *SHORT HYPOCOTYL 2* (*SHY2*) activity. New Phytol.

[66] Sellaro R, Pacín M, Casal JJ (2012). Diurnal dependence of growth responses to shade in *Arabidopsis*: role of hormone, clock, and light signaling. Mol Plant.

[67] Lu SX, Knowles SM, Andronis C, Ong MS, Tobin EM (2009). CIRCADIAN CLOCK ASSOCIATED1 and LATE ELONGATED HYPOCOTYL function synergistically in the circadian clock of *Arabidopsis*. Plant Physiol.

[68] Takase T, Nishiyama Y, Tanihigashi H, Ogura Y, Miyazaki Y, Yamada Y, Kiyosue T (2011). *LOV KELCH PROTEIN2* and *ZEITLUPE* repress *Arabidopsis* photoperiodic flowering under non-inductive conditions, dependent on *FLAVIN-BINDING KELCH REPEAT F-BOX1*. Plant J.

[69] Herrero E, Kolmos E, Bujdoso N, Yuan Y, Wang M, Berns MC, Uhlworm H, Coupland G, Saini R, Jaskolski M (2012). *EARLY FLOWERING4* recruitment of *EARLY FLOWERING3* in the nucleus sustains the *Arabidopsis* circadian clock. Plant Cell.

[70] Yakir E, Hilman D, Kron I, Hassidim M, Melamed-Book N, Green RM (2009). Posttranslational regulation of *CIRCADIAN CLOCK ASSOCIATED1* in the circadian oscillator of *Arabidopsis*. Plant Physiol.

[71] Song YH, Smith RW, To BJ, Millar AJ, Imaizumi T (2012). *FKF1* conveys timing information for *CONSTANS* stabilization in photoperiodic flowering. Science.

[72] Nishiyama R, Watanabe Y, Leyva-Gonzalez MA, Ha CV, Fujita Y, Tanaka M, Seki M, Yamaguchi-Shinozaki K, Shinozaki K, Herrera-Estrella L (2013). *Arabidopsis AHP2*, *AHP3*, and *AHP5* histidine phosphotransfer proteins function as redundant negative regulators of drought stress response. Proc Natl Acad Sci USA.

[73] González CV, Ibarra SE, Piccoli PN, Botto JF, Boccalandro HE (2012). Phytochrome B increases drought tolerance by enhancing ABA sensitivity in *Arabidopsis thaliana*. Plant, Cell Environ.

[74] Lin H, Han X, Feng X, Chen X, Lu X, Yuan Z, Li Y, Ye W, Yin Z (2022). Molecular traits and functional analysis of *Rapid Alkalinization Factors* (*RALFs*) in four *Gossypium* species. Int J Biol Macromol.

[75] Zhang JB, Wang XP, Wang YC, Chen YH, Luo JW, Li DD, Li XB (2020). Genome-wide identification and functional characterization of cotton (*Gossypium hirsutum*) *MAPKKK* gene family in response to drought stress. BMC Plant Biol.

[76] Alexieva V (2001). The effect of drought and ultraviolet radiation on growth and stress markers in *pea* and *wheat*. Plant Cell Environ.

