# Supplementary figures and images for: Structure and character analysis of cotton response regulator genes family reveals that GhRR7 responses to draught stress
Source: Biol Res. 2022 Aug 16;55:27. doi: 10.1186/s40659-022-00394-2 (PMC9380331; doi:10.1186/s40659-022-00394-2)

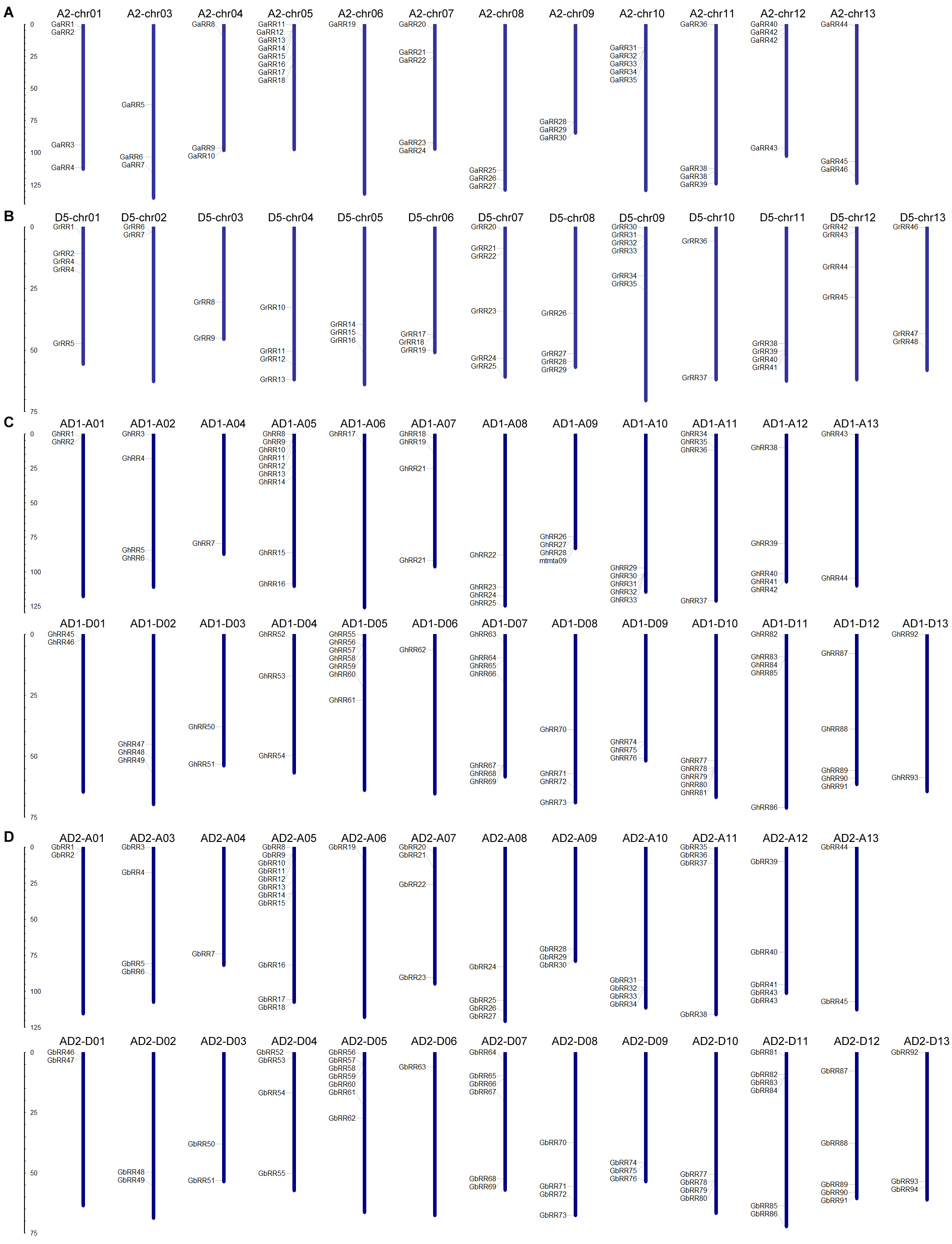

Supplement: Supplementary file 2 — Additional file 2: Figure S1. Chromosomal location of four Gossypium species. The scale on the left is inmega-bases. The gene ID on the right side of each chromosome corresponds to each RR gene's approximate locations. (A) Gossypium arboreum A-genome “A2”. (B) Gossypium raimondii D-sub genome “D5”. (C) Gossypium hirsutum genome “AD1”. (D) Gossypium barbadense genome “AD2”. [file 40659_2022_394_MOESM2_ESM.tif]

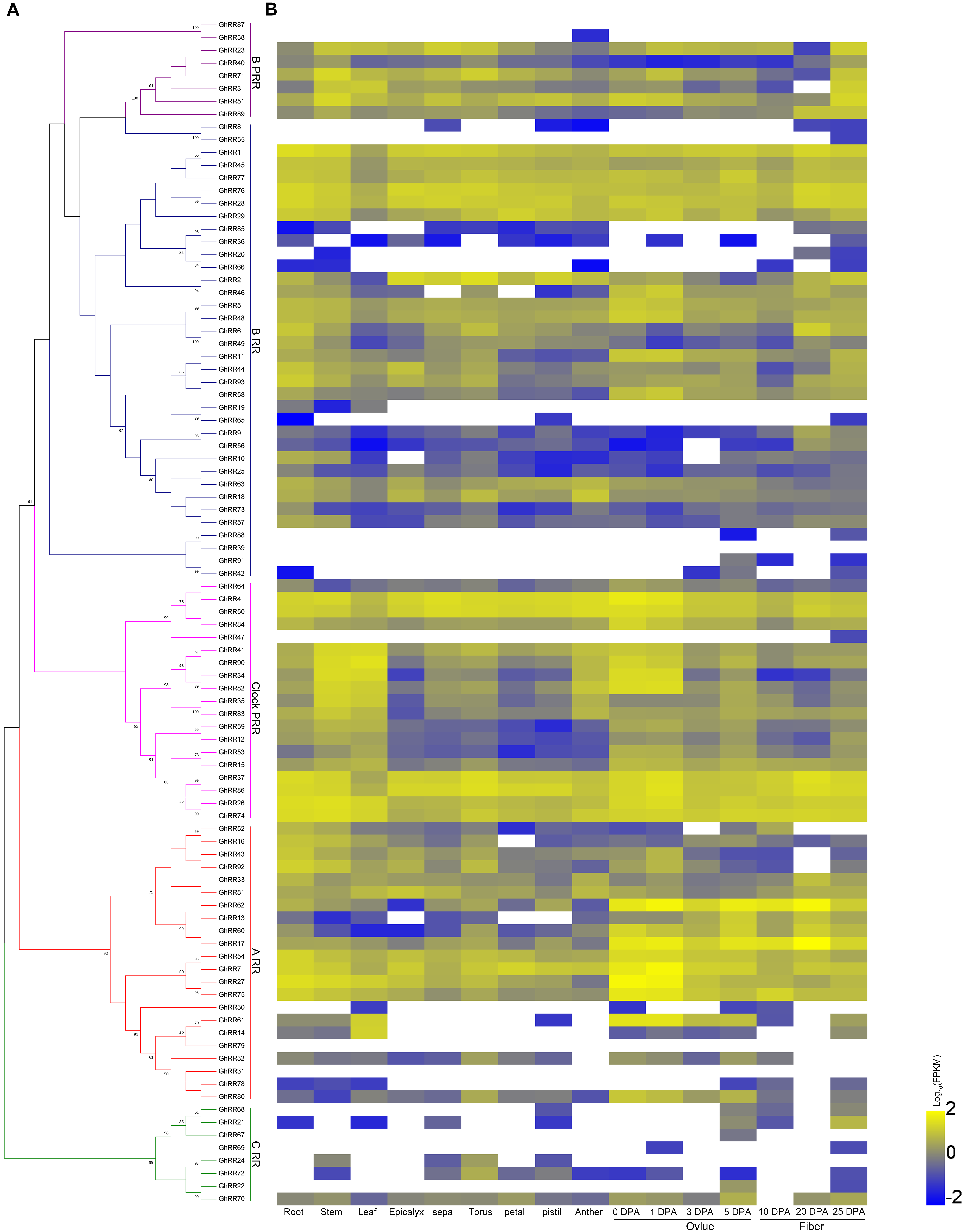

Supplement: Supplementary file 3 — Additional file 3: Figure S2. Analysis of RR genes expression pattern in different tissues. (A) Phylogenetic tree of GhRRs. (B) Expression pattern of GhRRs in different tissues. [file 40659_2022_394_MOESM3_ESM.tif]
